# Psychometrics, diagnostics and usability of Italian tools assessing behavioural and functional outcomes in neurological, geriatric and psychiatric disorders: a systematic review

**DOI:** 10.1007/s10072-022-06300-8

**Published:** 2022-08-06

**Authors:** Edoardo Nicolò Aiello, Alfonsina D’Iorio, Sonia Montemurro, Gianpaolo Maggi, Chiara Giacobbe, Valentina Bari, Gianluca Scotto Di Tella, Francesca Pischedda, Nadia Bolognini, Ildebrando Appollonio, Giorgio Arcara, Gabriella Santangelo

**Affiliations:** 1 PhD Program in Neuroscience, School of Medicine and Surgery, University of Milano-Bicocca, Via Cadore 48, 20900 Monza, Italy; 2 Department of Psychology, University of Campania “Luigi Vanvitelli”, Caserta, Italy; 3 IRCCS San Camillo Hospital, Venice, Italy; 4 Department of Psychology, University of Milano-Bicocca, Milano, Italy; 5 Neuropsychological Laboratory, IRCCS Istituto Auxologico Italiano, Milan, Italy; 6 Neurology Section, School of Medicine and Surgery, Milan Centre for Neuroscience (NeuroMI), University of Milano-Bicocca, Monza, Italy

**Keywords:** Behaviour, Functional outcome, Psychometrics, Diagnostics, Neurology, Geriatrics, Psychiatry

## Abstract

**Background:**

Psychometric instruments assessing behavioural and functional outcomes (BFIs) in neurological, geriatric and psychiatric populations are relevant towards diagnostics, prognosis and intervention. However, BFIs often happen not to meet methodological-statistical standards, thus lowering their level of recommendation in clinical practice and research. This work thus aimed at (1) providing an up-to-date *compendium* on psychometrics, diagnostics and usability of available Italian BFIs and (2) delivering evidence-based information on their level of recommendation.

**Methods:**

This review was pre-registered (PROSPERO ID: CRD42021295430) and performed according to PRISMA guidelines. Several psychometric, diagnostic and usability measures were addressed as outcomes. Quality assessment was performed via an ad hoc checklist, the Behavioural and Functional Instrument Quality Assessment.

**Results:**

Out of an initial *N* = 830 reports, 108 studies were included (*N* = 102 BFIs). Target constructs included behavioural/psychiatric symptoms, quality of life and physical functioning. BFIs were either self- or caregiver-/clinician-report. Studies in clinical conditions (including neurological, psychiatric and geriatric ones) were the most represented. Validity was investigated for 85 and reliability for 80 BFIs, respectively. Criterion and factorial validity testing were infrequent, whereas content and ecological validity and parallel forms were almost never addressed. Item response theory analyses were seldom carried out. Diagnostics and norms lacked for about one-third of BFIs. Information on administration time, ease of use and ceiling/floor effects were often unreported.

**Discussion:**

Several available BFIs for the Italian population do not meet adequate statistical-methodological standards, this prompting a greater care from researchers involved in their development.

**Supplementary Information:**

The online version contains supplementary material available at 10.1007/s10072-022-06300-8.

## Introduction

Psychometric instruments assessing behavioural dysfunctions (i.e. neuropsychiatric alterations within the affective, motivational, social and awareness dimensions) and functional outcomes (i.e. quality of life, functional independence and other aspects of physical *status*—*e.g.* sleep, pain or fatigue) in neurological, psychiatric and geriatric populations are relevant towards clinical phenotyping, prognosis and intervention practice [21]. Indeed, besides aiding clinical diagnosis, behavioural/functional instruments (BFIs) are often addressed as relevant to provide estimates of patients’ prognosis, being also adopted as clinical endpoints during interventional programs [21].

BFIs often present either self- or proxy-report (i.e. caregiver or healthcare professional) questionnaires, thus requiring sound psychometric and diagnostics, as well as evidence on clinical usability in target populations [6]. However, it has been highlighted that BFIs often do not meet methodological-statistical requirements, both when developed de novo and when adapted from a different language and culture [60]. Of note, such methodological-statistical lacks have been identified as detrimentally influencing the level of recommendation of a given tool both within clinical practice and research [13], [42].

Given the abovementioned premises, and based on the current knowledge on health measurement tools [58], this work aimed at assessing psychometrics, diagnostics and usability in neurological, geriatric and psychiatric populations of BFIs currently available in Italy, in order to (1) provide an up-to-date *compendium* on Italian BFIs designed for clinical and research aims in clinical populations and (2) deliver evidence-based information on the level of recommendation for Italian BFIs.

## Methods

### Search strategy

Preferred Reporting Items for Systematic Reviews and Meta-Analyses (PRISMA) guidelines were consulted [27]. This review was pre-registered on the International Prospective Register of Systematic Reviews (PROSPERO; ID: CRD42021295430; https://www.crd.york.ac.uk/prospero/display_record.php?ID=CRD42021295430).

A systematic literature search was performed on December 1, 2021 (no date limit set), entering the following search string into Scopus and PubMed databases: ( behavioural OR behavioral OR “quality of life” OR psychiatric OR psychopathological OR apathy OR depression OR anxiety OR qol OR mood OR “activities of daily living” OR “functional independence”) AND (validation OR validity OR standardization OR psychometric AND properties OR reliability OR version ) AND ( italian OR italy ) AND ( neurolog* OR neuropsych* OR cognitive ) AND ( questionnaire OR inventory OR tool OR instrument OR scale OR test OR interview OR checklist ). Fields of search were title, abstract and key words for Scopus whereas title and abstract for PubMed. Only peer-reviewed, full-text contributions written in English/Italian were considered. In addition, the reference lists of all relevant articles were further hand-searched in order to identify further eligible studies.

### Study eligibility criteria

Studies were evaluated for eligibility if they focused either on the psychometric/diagnostic/normative study of Italian/adapted to-Italian BFIs or their usability in healthy participants (HPs) and in patients with neurological/geriatric conditions or their proxies (e.g. caregivers). More specifically, eligible studies had to focus on (1) BFI psychometrics (i.e. validity and reliability) and (2) diagnostics (i.e. intrinsic (i.e. sensitivity, specificity) and post-test features (e.g. positive and negative predictive values and likelihood ratios)), or (3) norm derivation. Studies that did not aim at providing normative data were included only if at least one property among validity, reliability and sensitivity/specificity (or related metrics) was assessed.

Conference proceedings, letters to the Editor, commentaries, animal studies, single-case studies, reviews/meta-analyses, abstracts, research protocols, qualitative studies, opinion papers and studies on paediatric populations were excluded.

### Data collection and quality assessment

Screening stage was performed by two authors (E.N.A. and A.D.) and eligibility stage was performed by two other authors (G.M. and C.G.) via Rayyan (https://rayyan.qcri.org/ welcome); these stages were supervised by another author (V.B.).

Data extraction was performed by four independent Authors (S.M., G.S.D.T., V.B. and F.P.), whereas one independent author (E.N.A.) supervised this stage and checked extracted data.

Extracted outcomes included (1) sample size, (2) sample representativeness (geographic coverage, exclusion criteria), (3) participants’ demographics, (4) instruments adaptation procedures, (5) administration time, (6) validity metrics, (7) reliability metrics (including significant change measures), (8) measures of sensitivity and specificity, (9) metrics derived from sensitivity and specificity, (10) norming methods and (11) other psychometric/diagnostic properties (e.g. accuracy, acceptability rate, assessment of ceiling/floor effects, ease of use).

Formal quality assessment was performed by four Authors (S.M., G.S.D.T., V.B. and F.P.), and supervised by a further, independent one (E.N.A.). Quality assessment was performed for each BFI by developing two ad-hoc checklists, the Behavioural and Functional Instrument Quality Assessment-Normative Sample (BFIQA-NS) and the Behavioural and Functional Instrument Quality Assessment-Clinical Population (BFIQA-CP) (Supplemental Material 1), which were adapted from the Cognitive Screening Standardization Checklist (CSSC) [1]. Scores were “cumulatively” assigned for each BFI by evaluating all available studies on it among those included. Although some studies met the selection criteria, they did not answer some of the questions included in the CSSC, (e.g. diagnostic criteria for quality of life tools). In these cases, a group of items was scored as “non-applicable” and this was accounted, i.e. weighted, in the final score.

Both BFIQA-NS and BFIQA-CP total scores range from 0 to 50 and a given BFI was considered “statistically/methodologically sound” if scoring was ≥25, which means 50% out of the maximum. When more than one study focused on the same BFI in different populations, BFIQA scores were averaged (as both the BFIQA-NS and BFIQA-CP range from 0 to 50).

## Results

**Fig. 1:**
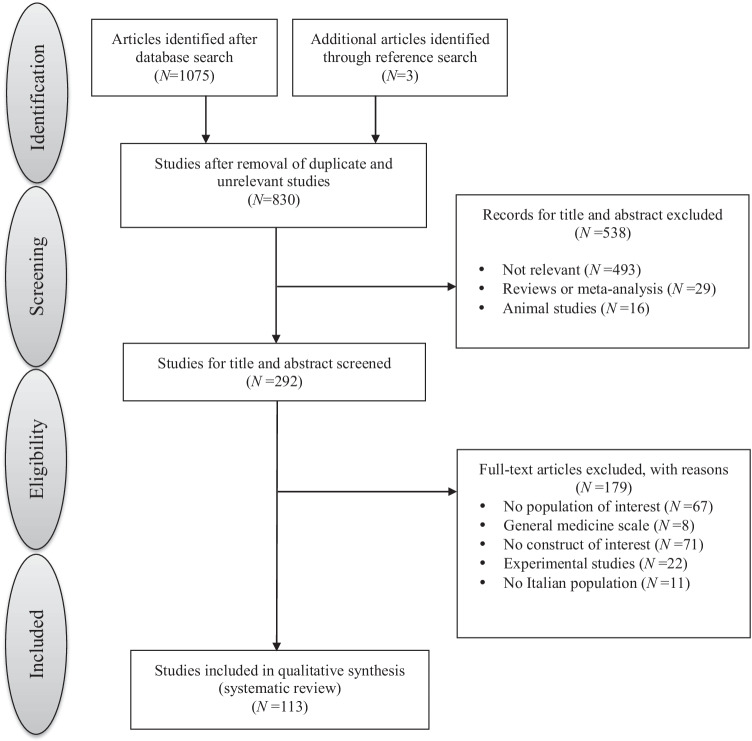
Study selection process according to PRISMA guidelines. Notes. Study selection process according to PRISMA guidelines. Adapted from Moher et al. (2009) (www.prisma-statement.org)

One-hundred and eighteen studies were included; study selection process according to PRISMA guidelines is shown in Figure 1.

The included BFIs (*N*=102), along with the summarization of the general clinical features and BFIQA scores are detailed in Table 1, while the most relevant psychometrics, diagnostics and usability evidence are described in Table 2 and Table 3. The full reference list of included studies is shown in the Supplementary File 2.

**Table 1:** Summarization of general clinical features of behavioural/functional instruments and BFIQA scores

								BFIQA	
								NS	CP
Behavioural/functional instrument, reference	*N*	Time	Self/proxy-report	NS	CP	Construct	Clinical conditions	/50	/50
AADS-I (Assessment for Adults with Developmental Disabilities) De Vreese et al. (2011)	1	20′	PRC PRHC		1	Behaviour (general)	Dementia in intellectual disability		23
ADI-12 (ALS Depression Inventory) Pain et al. [36]	1		SR		1	Behaviour (depression)	ALS		27
AES-C (Apathy Evaluation Scale—Clinician Version) Furneri et al. (2021)	1		PRHC	1	1	Behaviour (apathy)	AD, MCI	14	21
AES-I (Apathy Evaluation Scale) Borgi et al. [7]	1		PRC		1	Behaviour (apathy)	Geriatric conditions		24
AES-S (Apathy Evaluation Scale – Self-repost Version) Raimo et al. [44],Santangelo et al. [51]	2		SR		2	Behaviour (apathy)	MS, PD		30.5*
AFAST (Alzheimer’s Functional Assessment Tool) De Vreese et al. (2015)	1	10′	PRHC		1	Physical (FI)	Intellectual disability		22
ALS-CBSbi (ALS Cognitive Behavioral Screen) Tremolizzo et al. (2020)	1		PRC		1	Behaviour (general)	ALS		20
A-ONE (ADL-focused Occupation-based Neurobehavioral Evaluation) Cerone et al. (2021)	1		PRHC		1	Physical (FI)	Neurological conditions		15
AQ-D (Anosognosia Questionnaire-Dementia) Gambina et al. [17]	1		PRC PRHC		1	Behaviour (anosognosia)	AD		29
BANS-s (Bedford Alzheimer Nursing Severity scale) Bellelli et al. [4]	1		PRHC		1	Physical (FI)	AD, VaD		25
Barthel Index Tofani et al. (2019); Castiglia et al. (2017)	2		PRHC		2	Physical (FI)	Inpatients, PD		24*
BBI (Beaumont Behavioural Inventory) Iazzolino et al. [20]	1		PRC		1	Behaviour (general)	ALS	30	33
BDI-II (Beck Depression Inventory-II) Cuoco et al. [11],Sacco et al. (2015)	2		SR		2	Behaviour (depression)	MS, PSP		30.5*
BIS-15 (Barratt Impulsiveness Scale) Maggi et al. [28]	1		SR	1		Behaviour (impulsiveness)		31	
BPSD-DS (Behavioural and Psychological Symptoms of Dementia-Down Syndrome) Dekker et al. (2018)	1		PRC		1	Behaviour (general)	Down syndrome		24
BRIAN (Biological Rhythms Interview of Assessment in Neuropsychiatry) Moro et al. (2014)	1		PRHC	1	1	Multidimensional	Bipolar disorder	19	23
CADL-2 (Communication Activities of the Daily Living-II) Pigliautile et al. (2019)	1		PRHC	1	1	Behaviour (language)	Dementing conditions, MCI	17	19
CAMDEX (Cambridge Examination for Mental Disorders of the Elderly) Neri et al. (2001); Neri et al. (1998); Neri et al. (1992)	3		PRC PRHC	1	2	Behaviour (general)	AD	17	13.5*
CarerQol (Care-Related Quality of Life) Voormolen et al. [63]	1		PRC	1	1	QoL	Dementing conditions		29
CBA (Cognitive Behavioural Assessment) Bertolotti et al. (2015)	1		SR	1	1	Behaviour (attitudes towards treatments)	Mixed clinical populations	24	21
CES-D (Center for Epidemiological Studies Depression scale) Caracciolo et al. (2002)	1		SR		2	Behaviour (depression)	Inpatients, neurological conditions		17
CIRS (Clinical Insight Rating Scale) De Carolis et al. (2015)	1		PRHC		1	Behaviour (anosognosia)	Dementing conditions, MCI		11
CMT-HI (Charcot-Marie-Tooth Health Index) Pisciotta et al. (2020)	1		SR		1	Multidimensional	Charcot-Marie-Tooth disease		21
Coop/Wonca Pappalardo et al. [37]	1	< 5′	SR		1	QoL	MS		27
DAD-I (Disability Assessment Dementia scale) De Vreese et al. [12]	1		PRC PRHC		1	Physical (FI)	AD		25
DAFS (Direct Assessment of Functional Status) Zanetti et al. (1998)	1	41′	PRHC		1	Physical (FI)	AD		21
DAS (Dimensional Apathy Scale) Raimo et al. [46],Santangelo et al. [53],Santangelo et al. [54]	4		SR	1	3	Behaviour (apathy)	AD, ALS, MS	27	30*
DCPR-R (Diagnostic Criteria for Psychosomatic Research-Revised) Cosci et al. (2019)	1		PRHC		1	Behaviour (general)	Migraine		18
DHI-sf (Dizziness Handicap Inventory-short form) Tesio et al. (1999)	1	5′–10′	SR		1	Physical (FI)	Neurological conditions		15
DIDA-Q (Dual-task Impact on Daily-living Activities Questionnaire) Pedullà et al. [38]	1		SR		1	Physical (FI)	MS		25
DOLOPLUS Pickering et al. (2010)	1		PRHC		1	Physical (pain)	Aphasia		19
ELQ (Emotional Lability Questionnaire) Palmieri et al. (2008)	1		SR PRC	1	1	Behaviour (emotional lability)	ALS	12	18
eMSQOL-29-MS (Electronic format of Multiple Sclerosis Quality of Life-29) Rosato et al. [48]	1		PRHC		1	QoL	MS		37
Epi-QoL (Epilepsy-Quality of Life) Piazzini et al. [40]	1		SR		1	QoL	Epilepsy		28
ESS (Epworth Sleepiness Scale) Vignatelli et al. (2003)	1		SR		1	Physical (sleep)	Sleep disorders		24
FAM (Functional Assessment Measure) Tesio et al. (1998)	1	20′–30′	PRHC		1	Physical (FI)	TBI		19
FAS (Fibromyalgia Assessment Status) Iannuccelli et al. (2011)	1		SR		1	Physical (fatigue, sleep, pain)	Fibromyalgia		22
FBI (Frontal Behavioural Inventory) Milan et al. [29],Alberici et al. [2]	2		PRHC	1	3	Behaviour (general)	AD, FTD, VaD	17	29*
FIM (Functional Independence Measure) Lawton et al. (2006); Lundgren et al. (2005); Tesio et al. (2002); Franchignoni et al. (1995)	4		PRHC		3	Physical (FI)	Spinal cord injury, stroke		19*
FIQ (Fibromyalgia Impact Questionnaire) Iannuccelli et al. (2011)	1	5′	SR		1	Multidimensional	Fibromyalgia		22
FLSA (Functional Living Skills Assessment) Farina et al. (2010)	1	50′	PRHC	1	1	Physical (FI)	AD, VaD	23	24
GAI (Geriatric Anxiety Inventory) Rozzini et al. (2009)	1		SR PRHC		1	Behaviour (anxiety)	MCI		21
GAS (Geriatric Anxiety Scale) Picconi et al. (2018)	1		SR	1		Behaviour (anxiety)		23	
GDS (Geriatric Depression Scale) Rinaldi et al. (2003); Mondolo et al. [31],Galeoto et al. (2018)	3	8′–10′	SR PRHC	2	2	Behaviour (depression)	Major depressive disorder, PD	19.5*	23.5*
GDS-15 (Geriatric Depression Scale-15) Chiesi et al. (2017)	1		SR	1	1	Behaviour (depression)	Dementing conditions, MCI		15
GHS (Geriatric Handicap Scale) Verrusio et al. [62]	1		PRHC		1	Physical (FI)	Geriatric conditions		30
HADS (Hospital Anxiety and Depression Scale) Mondolo et al. [31]	1	< 5′	SR		1	Behaviour (depression, anxiety)	PD		27
HDRS (Hamilton Depression Rating Scale) Raimo et al. [45],Quaranta et al. [43],Mula et al. [33]	3	20′–30′	PRHC		1	Behaviour (depression)	Epilepsy, MS, stroke		27.33
HoNOS-RF (Health of the Nation Outcome Scales-Residential Facilities) Pedrini et al. (2017)	1		PRHC		1	Multidimensional	Psychiatric conditions		20
I-ASHA-FACS (Italian version of the American Speech-Language and Hearing Association-Functional Assessment of Communication Skills for adults) Muò et al. (2015)	1		PRHC	1	1	Behaviour (language)	Aphasia	18	21
IMD-ADL deficit (Impairment of Mental Decline-Activities of Daily Living deficit) Cucinotta et al. (1995)	1		PRC		1	Physical (FI)	Senile dementia of the Alzheimer type		16
IMD-Dem (Impairment of Mental Decline-Dementia) Cucinotta et al. (1995)	1		PRC		1	Physical (FI)	Dementing conditions		20
IPG-DepressionS (Intervista Psicogeriatrica-Depression Scales) Spagnoli et al. [57]	1		PRHC		1	Behaviour (depression)	Geriatric conditions		20
ISI (Insomnia Severity Index) Castronovo et al. (2016)	1		SR PRHC		1	Physical (sleep)	Sleep disorders		22
It-RMBPC (Italian version of the Revised Memory and Behaviour Problems Checklist) Ottoboni et al. (2019)	1		PRC		1	Behaviour (general)	AD		22
LIBRA index-MCI (Lifestyle for Brain Health index-Mild Cognitive Impairment) Franchini et al. (2019)	1		PRHC		2	Multidimensional	MCI, SCD		14.5*
MBI-C (Mild Behavioural Impairment Checklist) Elefante et al. (2019)	1		PRHC		1	Behaviour (general)	Dementing conditions, MCI		4
MDHI (Myotonic Dystrophy Health Index) Sansone et al. (2017)	1	14′	SR		1	Multidimensional	Myotonic dystrophy type I		24
MDQ (Misidentification Delusion Questionnaire) Perini et al. (2016)	1		PRC		1	Behaviour (psychosis)	AD, LBD		21
MDS-HC (Minimum DataSet for Home Care) Landi et al. [25]	1		PRHC		1	Physical (FI)	Geriatric conditions		15
MOS-HIV (Medical Outcomes Study-Human Immunodeficiency Virus) Tozzi et al. [59]	1	7′	SR		1	QoL	HIV		27
MPAI-4 (Mayo-Portland Adaptability Inventory-4) Cattelani et al. (2009)	1		PRC PRHC		1	Behaviour (general)	TBI		17
MSNQ (Multiple Sclerosis Neuropsychological Questionnaire) Migliore et al. (2021)	1		SR PRC		1	Behaviour (general)	MS		21
MSQOL-29 (Multiple Sclerosis Quality Of Life-29) Rosato et al. [47]	1	10′	SR		1	QoL	MS		27
MSQOL-54 (Multiple Sclerosis Quality Of Life-54) Solari et al. [56]	1		PRHC		1	QoL	MS		36
Multidimensional Assessment Provinciali et al. (1999)	1	70′	SR PRHC		1	Multidimensional	MS		19
NDDI-E (Neurological Disorders Depression Inventory for Epilepsy) Mula et al. (2012)	1		SR		1	Behaviour (depression)	Epilepsy		23
NPI-NH (Neuropsychiatric Inventory-Nursing Home Version) Baranzini et al. [3]	1		PRHC		1	Behaviour (general)	Geriatric conditions		27
NMSQuest (Non-Motor Symptoms Questionnaire) Cova et al. [9]	1		SR PRC		1	Multidimensional	PD		21
NMSS (Non-Motor Symptoms Scale) Cova et al. [9]	1		PRHC		1	Multidimensional	PD		25
NOPPAIN (Non-Communicative Patient’s Pain Assessment Instrument) Ferrari et al. [15]	1		PRHC	1	1	Physical (pain)	Dementing conditions	22	29
NPI (Neuropsychiatric Inventory) De Carolis et al. (2015)	1		PRHC		1	Behaviour (general)	Dementing conditions, MCI		12
OR-PAS (Observer-Rated version of the Parkinson Anxiety Scale) Santangelo et al. [53]	1		PRHC		1	Behaviour (anxiety)	PD		31
PAIC (Pain Assessment in Impaired Cognition) Kunz et al. (2021)	1		PRHC		1	Physical (pain)	Huntington’s disease		9
PAINAD (Pain Assessment in Advanced Dementia) Costardi et al. [8],Mosele et al. [32]	2		SR		1	Physical (pain)	Dementing conditions		31*
PASS (Progressive Aphasia Severity Scale) Petrillo et al. (2021)	1	10′	PRC		1	Physical (FI)	PPA		8
PDSS-2 (Parkinson Disease Sleep Scale 2) Arnaldi et al. (2016)	1		SR		1	Physical (sleep)	PD		24
PRIMUS (Patient-Reported Indices for Multiple Sclerosis) McKenna et al. (2010)	1		SR		1	Multidimensional	MS		24
PSDRS (Post-Stroke Depression Rating Scale) Quaranta et al. [43]	1	15′	PRHC		1	Behaviour (depression)	Stroke		23
PSP-QoL (Progressive Supranuclear Palsy-Quality of Life) Picillo et al. [41]	1		SR		1	QoL	PSP		29
QLQA (Quality of Life Questionnaire for Aphasics) Spaccavento et al. (2014)	1		PRHC		2	QoL	Aphasia		18
QoL-AD (Quality of Life in Alzheimer’s Disease) [5]	1		PRHC		1	QoL	Dementing conditions		29
QOL-DyS (Quality of Life in the Dysarthric Speaker) Piacentini et al. [39]	1		SR		1	QoL	Dysarthria		30
QOLIBRI (Quality of Life after Brain Injury) Giustini et al. [19],Formisano et al. [16]	2		SR PRC PRHC		2	QoL	TBI		25.5*
QOLIE-31 (Quality of Life in Epilepsy Inventory-31) Cramer et al. (1998); Beghi et al. (2005)	2		PRHC		2	QoL	Epilepsy		17.5*
QSMDPD (Questionnaire on Sleep and Mental Disorders in Parkinson’s Disease) Pacchetti et al. (2004)	1		SR PRHC		1	Multidimensional	PD		16
SADBD*-*STROKE (Structured Assessment of Depression in Brain Damaged individuals-stroke) Monaco et al. (2005)	1	45′–60′	PRHC		1	Behaviour (depression)	Stroke		18
SAS-6 (Starkstein Apathy Scale-6) Garofalo et al. (2020)	1		PRHC	1		Behaviour (apathy)		23	
SAS-I (Starkstein Apathy Scale) Garofalo et al. (2020)	1		PRHC	1		Behaviour (apathy)		28	
SDRS (Social Dysfunction Rating Scale) Lozupone et al. (2018)	1		PRHC	1	1	Behaviour (social)	Psychiatric conditions	21	18
SHAPS (Snaith-Hamilton Pleasure Scale) Martino et al. (2018); Santangelo et al. (2009)	2		SR	2	1	Behaviour (anhedonia)	PD	24	22*
SIB-Social Interaction Subscale (Severe Impairment Battery) Pippi et al. (1999)	1		PRHC		1	Behaviour (social)	Dementing conditions		19
SIS 3.0 (Stroke Impact Scale 3.0) Vellone et al. [61]	1		PRHC		1	QoL	Stroke		33
Sleep questionnaire Manni et al. (2013)	1		SR PRC PRHC		1	Physical (sleep)	AD		17
SLOF (Specific Level Of Functioning) Montemagni et al. (2015)	1		PRC		1	Physical (FI)	Schizophrenia spectrum disorders		12
STAI (State-Trait Anxiety Inventory) Santangelo et al. [53],Siciliano et al. [55],Ilardi et al. [24]	3		SR	1	2	Behaviour (anxiety)	ALS, MS	20	26.5*
STAI-S (State-Trait Anxiety Inventory-State Anxiety Subscale) Annunziata et al. (2016)	1		SR	1		Behaviour (anxiety)		15	
STICSA (State-Trait Inventory for Cognitive and Somatic Anxiety) Balsamo et al. (2015)	1		SR	1		Behaviour (anxiety)		20	

*, mean of the total score for normal and clinical population respectively, when the same instrument was used in more than one study; *AD*, Alzheimer’s disease; *ALS*, amyotrophic lateral sclerosis; *BFIQA*, Behavioural and Functional Instrument Quality Assessment; *CP*, clinical population; *FI*, functional independence; *FTD*, frontotemporal dementia; *LBD*, Lewy body dementia; *MCI*, mild cognitive impairment; *MS*, multiple sclerosis; *NS*, normative sample; *PD*, Parkinson’s disease; *PPA*, primary progressive aphasia; *PRC*, proxy-report (caregiver); *PRHC*, proxy-report (health-care professional); *PSP*, progressive supranuclear palsy; *QoL*, quality of life; *SCD*, subjective cognitive decline; *SR*, self-report; *TBI*, traumatic brain injury; *VaD*, vascular dementia

**Table 2:** Summarization of main psychometrics evidence

		Validity					Reliability				
BFI, reference	*N*	Convergent validity	Criterion validity	Divergent validity	Factorial structure	Face validity	Test–retest reliability	Inter-rater reliability	Internal consistency	Parallel forms	Reliable change index
AADS-I De Vreese et al. (2011)	1	Y	N	N	N	N	N	Y	Y	N	N
ADI-12 Pain et al. [36]	1	Y	N	N	Y	N	N	N	Y	N	N
AES-C Furneri et al. (2021)	1	Y	N	Y	Y	N	Y	Y	Y	N	N
AES-I Borgi et al. [7]	1	Y	N	Y	Y	N	Y	N	Y	N	N
AES-S Raimo et al. [44],Santangelo et al. [51]	2	Y	Y	Y	Y	N	Y	N	Y	N	N
AFAST De Vreese et al. (2015)	1	Y	N	Y	N	N	Y	N	Y	N	N
ALS-CBSbi Tremolizzo et al. (2020)	1	N	N	N	N	N	N	N	N	N	N
A-ONE Cerone et al. (2021)	1	Y	N	N	N	N	Y	Y	Y	N	N
AQ-D Gambina et al. [17]	1	N	Y	N	Y	N	N	Y	Y	N	N
BANS-s Bellelli et al. [4]	1	Y	N	Y	N	N	Y	Y	N	N	N
Barthel Index Tofani et al. (2019); Castiglia et al. (2017)	2	Y	N	N	Y	N	Y	Y	Y	N	N
BBI Iazzolino et al. [20]	1	Y	Y	Y	N	Y	N	N	Y	N	N
BDI-II Cuoco et al. [11],Sacco et al. (2015)	2	Y	Y	Y	Y	Y	N	N	Y	N	N
BIS-15 Maggi et al. [28]	1	Y	N	Y	Y	N	N	N	Y	N	N
BPSD-DS Dekker et al. (2018)	1	N	N	N	N	Y	N	N	Y	N	N
BRIAN Moro et al. (2014)	1	Y	N	Y	N	N	Y	N	N	N	N
CADL-2 Pigliautile et al. (2019)	1	N	N	N	N	N	N	N	Y	N	N
CAMDEX Neri et al. (2001); Neri et al. (1998); Neri et al. (1992)	3	Y	Y	N	N	N	N	Y	N	N	N
CarerQol Voormolen et al. [63]	1	Y	Y	N	N	N	N	N	N	N	N
CBA Bertolotti et al. (2015)	1	Y	Y	Y	Y	N	Y	N	Y	N	N
CES-D Caracciolo et al. (2002)	1	Y	Y	N	N	N	N	N	N	N	N
CIRS De Carolis et al. (2015)	1	N	N	N	N	N	N	N	N	N	N
CMT-HI Pisciotta et al. (2020)	1	Y	N	N	N	N	Y	N	N	N	N
Coop/Wonca Pappalardo et al., [37]	1	Y	N	N	N	N	N	Y	Y	N	N
DAD-I De Vreese et al. [12]	1	Y	N	Y	N	N	Y	N	Y	N	N
DAFS Zanetti et al. (1998)	1	N*	Y	N	N	N	N	N	N	N	N
DAS Raimo et al. (2019); Santangelo et al. [53],Santangelo et al. [54]	4	Y	Y	Y	Y	N	N	N	Y	N	N
DCPR-R Cosci et al. (2019)	1	N	Y	N	N	N	N	N	N	N	N
DHI-sf Tesio et al. (1999)	1	N	N	N	Y	N	Y	N	N	N	N
DIDA-Q Pedullà et al. [38]	1	Y	N	Y	Y	N	Y	N	Y	N	N
DOLOPLUS Pickering et al. (2010)	1	N	N	N	N	N	Y	Y	N	N	N
ELQ Palmieri et al. (2008)	1	Y	N	N	N	N	N	N	N	N	N
eMSQOL-29-MS Rosato et al. (2018)	1	N	Y	N	Y	N	Y	N	Y	N	Y
Epi-QoL Piazzini et al. [40]	1	N	N	N	N	N	Y	N	Y	N	N
ESS Vignatelli et al. (2003)	1	N*	N	N	N	N	N	N	N	N	N
FAM Tesio et al. (1998)	1	N	N	N	N	N	N	N	Y	Y	N
FAS Iannuccelli et al. (2011)	1	Y	N	N	N	N	N	N	N	N	N
FBI Milan et al. [29],Alberici et al. [2]	2	Y	Y	Y	Y	N	Y	Y	Y	N	N
FIM Lawton et al. (2006); Lundgren et al. (2005); Tesio et al. (2002); Franchignoni et al. (1995)	4	N*	N	N	Y	N	N	N*	N	N	N
FIQ Iannuccelli et al. (2011)	1	N*	N	N	N	N	N	N	N	N	N
FLSA Farina et al. (2010)	1	Y	N	N	N	N	Y	Y	Y	N	N
GAI Rozzini et al. (2009)	1	N*	Y	N	N	N	Y	Y	Y	N	N
GAS Picconi et al. (2018)	1	Y	N	Y	Y	N	N	N	Y	N	N
GDS Rinaldi et al. (2003); Mondolo et al. [31],Galeoto et al. (2018)	3	N	Y	N*	Y	N	Y	Y	Y	N	N
GDS-15 Chiesi et al. (2017)	1	N	N	N	N	N	N	N	N	N	N
GHS Verrusio et al. [62]	1	Y	N	N	N	N	N	N	Y	N	N
HADS Mondolo et al. [31]	1	N	Y	N*	N*	N	N	N	N*	N	N
HDRS Raimo et al. [45],Quaranta et al. [43],Mula et al. [33]	3	Y	Y	Y	N	N	N	N	Y	N	N
HoNOS-RF Pedrini et al. (2017)	1	Y	N	N	N	N	N	Y	Y	N	N
I-ASHA-FACS Muò et al. (2015)	1	Y	N	N	N	N	Y	Y	Y	N	N
IMD-ADL deficit Cucinotta et al. (1995)	1	N	N	N	N	N	Y	Y	N	N	N
IMD-Dem Cucinotta et al. (1995)	1	Y	N	N	N	N	N	N	N	N	N
IPG-DepressionS Spagnoli et al. [57]	1	N	Y	N	N	N	Y	Y	N	N	N
ISI Castronovo et al. (2016)	1	N	Y	N	Y	N	N	N	Y	N	N
It-RMBPC Ottoboni et al. (2019)	1	N	Y	N	Y	Y	Y	N	Y	N	N
LIBRA*-*index Franchini et al. (2019)	1	N	N	N	N	N	N	N	N	N	N
MBI-C Elefante et al*.* (2019)	1	N	N	N	N	N	N	N	N	N	N
MDHI Sansone et al. (2017)	1	N	Y	N	N	N	Y	N	Y	N	N
MDQ Perini et al. (2016)	1	N	Y	N	N	N	N	N	N	N	N
MDS-HC Landi et al. [25]	1	N	Y	N	N	N	N*	N*	N	N	N
MOS-HIV Tozzi et al. [59]	1	N*	N	N*	N	N	N	N	N*	N	N
MPAI-4 Cattelani et al. (2009)	1	N	N	N	N	N	N	Y	Y	N	N
MSNQ Migliore et al. (2021)	1	Y	Y	N	Y	N	N	N	N	N	N
MSQOL-29 Rosato et al. (2015)	1	N	N	N	Y	N	N	N	N	N	N
MSQOL-54 Solari et al. [56]	1	Y	Y	Y	N	Y	N	N	Y	N	N
Multidimensional Assessment Provinciali et al. (1999)	1	N	N	N	Y	N	N	N	N	N	N
NDDI-E Mula et al. (2012)	1	N	N	N	N	N	N	N	Y	N	N
NPI-NH Baranzini et al. [3]	1	Y	N	N	Y	N	Y	Y	Y	N	N
NMSQuest Cova et al. [9]	1	Y	N	Y	N	N	Y	N	Y	N	N
NMSS Cova et al. [9]	1	Y	N	Y	N	N	Y	N	Y	N	N
NOPPAIN Ferrari et al. [15]	1	Y	Y	N	N	N	N	Y	N	N	N
NPI De Carolis et al. (2015)	1	N*	N	N	N	N	N	N	N*	N	N
OR-PAS Santangelo et al. [53]	1	Y	N	Y	N	N	N	N	Y	N	N
PAIC Kunz et al. (2021)	1	N	N	N	N	N	N	N	N	N	N
PAINAD Costardi et al. [8],Mosele et al. [32]	2	Y	Y	Y	N	N	Y	Y	Y	N	N
PASS Petrillo et al. (2021)	1	N	N	N	N	N	N	N	N	N	N
PDSS-2 Arnaldi et al. (2016)	1	Y	N	N	Y	N	Y	N	Y	N	N
PRIMUS McKenna et al. (2010)	1	Y	N	N	Y	Y	Y	N	Y	N	Y
PSDRS Quaranta et al. [43]	1	N	N	N	N	N	N	N	Y	N	N
PSP-QoL Picillo et al. [41]	1	Y	N	Y	N	N	N	N	Y	N	N
QLQA Spaccavento et al. (2014)	1	Y	N	Y	Y	N	N	N	Y	N	N
QoL-AD [5]	1	N	N	N	N	Y	Y	N	Y	N	N
QOL-DyS Piacentini et al. [39]	1	Y	Y	N	N	N	Y	N	Y	N	N
QOLIBRI Giustini et al. [19],Formisano et al., [16]	2	N	Y	N	N	N	Y	N	Y	N	N
QOLIE-31 Cramer et al. (1998); Beghi et al. (2005)	2	Y	Y	Y	Y	N	N	N	Y	N	N
QSMDPD Pacchetti et al. (2004)	1	N	N	N	N	N	N	N	N	N	N
SADBD-STROKE Monaco et al. (2005)	1	Y	N	N	N	N	Y	Y	Y	N	N
SAS-6 Garofalo et al. [18]	1	N	N	N	N	Y	N	N	N	N	N
SAS-I Garofalo et al. [18]	1	Y	N	Y	N	N	N	N	Y	N	N
SDRS Lozupone et al. (2018)	1	N	N	N	Y	N	N	N	Y	N	N
SHAPS Martino et al. (2018); Santangelo et al. (2009)	2	Y	N	Y	Y	N	Y	N	Y	N	N
SIB-Social Interaction Subscale Pippi et al. (1999)	1	N	N	N	N	N	Y	Y	N	N	N
SIS 3.0 Vellone et al. [61]	1	N	Y	Y	Y	N	Y	N	Y	N	N
Sleep questionnaire Manni et al. (2013)	1	N	N	N	N	N	N	N	Y	N	N
SLOF Montemagni et al. (2015)	1	N*	N	N*	N*	N	N	N	N*	N	N
STAI Santangelo et al. [53] Siciliano et al. [55],Ilardi et al. [24]	3	N	N	Y	Y	N	N	N	Y	N	N
STAI-S Annunziata et al. (2016)	1	N*	N	N*	N	N	N	N	N*	N	N
STICSA Balsamo et al. (2015)	1	Y	N	Y	Y	N	N	N	Y	N	N

*Y*, explicitly investigated within reference studies; N*, not investigated within reference studies but retrievable from different ones; *N*, not investigated within reference studies. *AADS-I*, Assessment for Adults with Developmental Disabilities-Italian version; *ADI-12*, ALS Depression Inventory-12; *AES-C*, Apathy Evaluation Scale-Clinician version; *AES-I*, Apathy Evaluation Scale-Italian version; *AES-S*, Apathy Evaluation Scale-Self report; *AFAST*, Alzheimer’s Functional Assessment Tool; *ALS-CBSbi*, ALS Cognitive Behavioural Screen-Behavioural Inventory; *A-ONE*, Activities of daily living-focused Occupation-based Neurobehavioural Evaluation; Aphasia; *AQ-D*, Anosognosia Questionnaire-Dementia; *BANS-s*, Bedford Alzheimer Nursing Severity scale; *BBI*, Beaumont Behavioural Inventory; *BDI-II*, Beck Depression Inventory-II; *BFI*, Behavioural/Functional Instrument; *BIS-15*, Barratt Impulsiveness Scale; *BPSD-DS*, Behavioural and Psychological Symptoms of Dementia-Down Syndrome; *BRIAN*, Biological Rhythms Interview of Assessment in Neuropsychiatry; *CADL-2*, Communication Activities of the Daily Living-II; *CAMDEX*, Cambridge Examination for Mental Disorders of the Elderly; *CAMDEX-I*, Cambridge Examination for Mental Disorders of the Elderly-Interview; *CarerQol*, Care-Related Quality of Life; *CBA*, Cognitive Behavioural Assessment; *CES-D*, Center for Epidemiological Studies Depression scale; *CIRS*, Clinical Insight Rating Scale; *CMT-HI*, Charcot-Marie-Tooth Health Index; *CNS*, Central nervous System; *DAD-I*, Disability Assessment Dementia scale-Italian version; *DAFS*, Direct Assessment of Functional Status; *DAS*, Dimensional Apathy Scale; *DCPR-R*, Diagnostic Criteria for Psychosomatic Research-Revised; *DHI-sf*, Dizziness Handicap Inventory-short form; *DIDA-Q*, Dual-task Impact on Daily-living Activities Questionnaire; *DSM-V*, Diagnostic and Statistical Manual of mental disorders-Fifth Edition; *ELQ*, Emotional Lability Questionnaire; *eMSQOL-29-MS*, Electronic format of Multiple Sclerosis Quality of Life-29; *Epi-QoL*, Epilepsy-Quality of Life; *ESS*, Epworth Sleepiness Scale; *FAM*, Functional Assessment Measure; *FAS*, Fibromyalgia Assessment Status; *FBI-AD*, Frontal Behavioural Inventory-Alzheimer Disease; *FIM*, Functional Independence Measure; *FIQ*, Fibromyalgia Impact Questionnaire; *FLSA*, Functional Living Skills Assessment; *FS*, Factorial Structure; *FV*, Face Validity; *GAI*, Geriatric Anxiety Inventory; *GAS*, Geriatric Anxiety Scale; *GDS*, Geriatric Depression Scale; *GDS-15*, Geriatric Depression Scale-15; *GHS*, Geriatric Handicap Scale; *HADS*, Hospital Anxiety and Depression Scale; *HDRS*, Hamilton Depression Rating Scale; *HoNOS-RF*, Health of the Nation Outcome Scales-Residential Facilities; *I-ASHA-FACS*, Italian version of the American Speech-Language and Hearing Association-Functional Assessment of Communication Skills for adults; *IC*, Internal Consistency; *IMD-ADLdeficit*, Impairment of Mental Decline-Activities of Daily Living deficit; *IMD-Dem*, Impairment of Mental Decline-Dementia; *IPG-DepressionS*, Intervista Psicogeriatrica-Depression Scales; *ISI*, Insomnia Severity Index; *It-RMBPC*, Italian version of the Revised Memory and Behaviour Problems Checklist; *LIBRA-index-MCI*, Lifestyle for Brain Health index-Mild Cognitive Impairment; *MBI*, Mild Behavioural Impairment; *MBI-C*, Mild Behavioural Impairment Checklist; *MDHI*, Myotonic Dystrophy Health Index; *MDQ*, Misidentification Delusion Questionnaire; *MDS-HC*, Minimum DataSet for Home Care; *MINI*, Mini-International Neuropsychiatric Interview; *MOS-HIV*, Medical Outcomes Study-Human Immunodeficiency Virus; *MPAI-4*, Mayo-Portland Adaptability Inventory-4; *MSNQ*, Multiple Sclerosis Neuropsychological Questionnaire; *MSQOL-29*, Multiple Sclerosis Quality Of Life-29; *MSQOL-54*, Multiple Sclerosis Quality Of Life-54; *NDDI-E*, Neurological Disorders Depression Inventory for Epilepsy; *NMSQuest*, Non-Motor Symptoms Questionnaire; *NMSS*, Non-Motor Symptoms Scale; *NOPPAIN*, Non-Communicative Patient’s Pain Assessment Instrument; *NPI*, Neuropsychiatric Inventory; *OR-PAS*, Observer-Rated version of the Parkinson Anxiety Scale; *PAIC*, Pain Assessment in Impaired Cognition; *PAINAD*, Pain Assessment in Advanced Dementia; *PASS*, Progressive Aphasia Severity Scale; *PDSS-2*, Parkinson Disease Sleep Scale 2; *PF*, Parallel Forms; *PPA*, Primary Progressive Aphasia; *PRIMUS*, Patient-Reported Indices for Multiple Sclerosis; *PSDRS*, Post-Stroke Depression Rating Scale; *PSP-QoL*, Progressive Supranuclear Palsy-Quality of Life; *QLQA*, Quality of Life Questionnaire for Aphasics; *QoL-AD*, Quality of Life in Alzheimer’s Disease; *QOL-DyS*, Quality of Life in the Dysarthric Speaker; *QOLIBRI*, Quality of Life after Brain Injury; *QOLIE-31*, Quality of Life in Epilepsy Inventory-31 dimensions; *QSMDPD*, Questionnaire on Sleep and Mental Disorders in Parkinson’s Disease; *RCI*, Reliable Change Index; *SADBD-STROKE*, Structured Assessment of Depression in Brain Damaged individuals-stroke; *SDRS*, Social Dysfunction Rating Scale; *SHAPS*, Snaith-Hamilton Pleasure Scale; *SIB*, Severe Impairment Battery; *SIS 3.0*, Stroke Impact Scale 3.0; *SLOF*, Specific Level Of Functioning; *STAI*, State-Trait Anxiety Inventory; *STAI-S*, State-Trait Anxiety Inventory-State Anxiety Subscale; *STICSA*, State-Trait Inventory for Cognitive and Somatic Anxiety

**Table 3:** Summarization of main diagnostics and usability evidence

			Diagnostics									Usability		
BFI, reference	*N*	IRT	Sensitivity	Specificity	PPV	NPV	LR +	LR-	AUC	Cut-off	Back Translation	Acceptability	Ease of use	Ceiling/floor effects
AADS-I De Vreese et al. (2011)	1	N	N	N	N	N	N	N	N	N	Y	N	N	N
ADI-12 Pain et al. [36]	1	N	Y	Y	N	N	N	N	Y	Y	Y	N	N	N
AES-C Furneri et al. (2021)	1	N	N	N	N	N	Y	N	N	N	Y	N	N	N
AES-I Borgi et al. [7]	1	N	N	N	N	N	N	N	N	N	Y	N	N	N
AES-S Raimo et al. [44],Santangelo et al. [51]	2	N	Y	Y	Y	Y	N	N	Y	Y	N	Y	N	Y
AFAST De Vreese et al. (2015)	1	N	N	N	N	N	N	N	N	N	Y	N	N	N
ALS-CBSbi Tremolizzo et al. (2020)	1	N	N	N	N	N	N	N	N	N*	Y	Y	N	N
A-ONE Cerone et al. (2021)	1	N	N	N	N	N	N	N	N	N	N	N	N	N
AQ-D Gambina et al. [17]	1	N	Y	Y	N	N	N	N	Y	Y	Y	Y	N	N
BANS-s Bellelli et al. [4]	1	N	N	N	N	N	N	N	N	N	N	N	N	Y
Barthel Index Tofani et al. (2019); Castiglia et al. (2017)	2	N	Y	Y	Y	Y	Y	Y	Y	Y	N*	N	N	Y
BBI Iazzolino et al. [20]	1	N	Y	Y	N	N	N	N	Y	Y	Y	N	N	N
BDI-II Cuoco et al. [11],Sacco et al. (2015)	2	N	Y	Y	Y	Y	N	N	Y	Y	N	Y	N	Y
BIS-15 Maggi et al. [28]	1	Y	N	N	N	N	N	N	N	Y	N*	Y	N	Y
BPSD-DS Dekker et al. (2018)	1	N	N	N	N	N	N	N	N	N	N	N	N	N
BRIAN Moro et al. (2014)	1	N	Y	Y	Y	Y	N	N	Y	Y	Y	N	N	N
CADL-2 Pigliautile et al. (2019)	1	N	N	N	N	N	N	N	Y	N	Y	N	N	N
CAMDEX Neri et al. (2001); Neri et al. (1998); Neri et al. (1992)	3	N	Y	Y	N	N	N	N	N	Y	N	N	N	N
CarerQol Voormolen et al. [63]	1	N	N/A	N/A	N/A	N/A	N/A	N/A	N/A	N/A	N*	N	N	N
CBA Bertolotti et al. (2015)	1	N	N	N	N	N	N	N	N	N	Y	N	N	N
CES-D Caracciolo et al. (2002)	1	N	Y	Y	Y	Y	Y	N	N	Y	N	N	N	N
CIRS De Carolis et al. (2015)	1	N	N	N	N	N	N	N	N	N	N	N	N	N
CMT-HI Pisciotta et al. (2020)	1	N	N	N	N	N	N	N	N	N	Y	Y	N	N
Coop/Wonca Pappalardo et al. [37]	1	N	N/A	N/A	N/A	N/A	N/A	N/A	N/A	N/A	Y	N	N	N
DAD-I De Vreese et al. [12]	1	N	N	N	N	N	N	N	N	N	Y	N	N	N
DAFS Zanetti et al. (1998)	1	N	N	N	N	N	N	N	N	N	N	N	N	N
DAS Raimo et al. (2019); Santangelo et al. [53],Santangelo et al. [54]	4	N	Y	Y	Y	Y	N	N	Y	Y	Y	Y	N	Y
DCPR-R Cosci et al. (2019)	1	N	N	N	N	N	N	N	N	N	N	N	N	N
DHI-sf Tesio et al. (1999)	1	Y	N	N	N	N	N	N	N	N	N	N	N	N
DIDA-Q Pedullà et al. [38]	1	N	N	N	N	N	N	N	N	N	N	Y	N	N
DOLOPLUS Pickering et al. (2010)	1	N	N/A	N/A	N/A	N/A	N/A	N/A	N/A	N/A	Y	Y	N	N
ELQ Palmieri et al. (2008)	1	N	N	N	N	N	N	N	N	N	Y		N	N
eMSQOL-29*-*MS Rosato et al. (2018)	1	N	N/A	N/A	N/A	N/A	N/A	N/A	N/A	N/A	N	Y	Y	Y
Epi-QoL Piazzini et al. [40]	1	N	N/A	N/A	N/A	N/A	N/A	N/A	N/A	N/A	N	N	N	Y
ESS Vignatelli et al. (2003)	1	N	Y	Y	Y	Y	Y	Y	N	Y	Y	Y		
FAM Tesio et al. (1998)	1	Y	N	N	N	N	N	N	N	N	N	N	Y	Y
FAS Iannuccelli et al. (2011)	1	N	N/A	N/A	N/A	N/A	N/A	N/A	N/A	N/A	N		Y	N
FBI Milan et al. [29],Alberici et al. [2]	2	N	Y	Y	N	N	N	N	Y	Y	Y	N	N	N
FIM Lawton et al. (2006); Lundgren et al. (2005); Tesio et al. (2002); Franchignoni et al. (1995)	4	Y	N	N	N	N	N	N	N	N	N*	N	N	Y
FIQ Iannuccelli et al. (2011)	1	N	N/A	N/A	N/A	N/A	N/A	N/A	N/A	N/A	N	N	N*	N
FLSA Farina et al*.* (2010)	1	N	Y	Y	N	N	N	N	N	Y	N	N	Y	N
GAI Rozzini et al. (2009)	1	N	N	N	N	N	N	N	N	N	Y	N	N	N
GAS Picconi et al. (2018)	1	N	N	N	N	N	N	N	N	N	N*	N	N	N
GDS Rinaldi et al. (2003); Mondolo et al. [31],Galeoto et al. (2018)	3	N	Y	Y	Y	Y	Y	Y	Y	Y	N	Y	N	N
GDS-15 Chiesi et al. (2017)	1	Y	N	N	N	N	N	N	N	N	N	N	N	N
GHS Verrusio et al. [62]	1	N	Y	Y	N	N	N	N	Y	Y	N	N	N	N
HADS Mondolo et al. [31]	1	N	Y	Y	Y	Y	N	N	Y	Y	N*	N	N	N
HDRS Raimo et al. [45],Quaranta et al. [43],Mula et al. [33]	3	N	Y	Y	Y	Y	N	N	Y	Y	N	Y	N	Y
HoNOS-RF Pedrini et al. (2017)	1	N	N	N	N	N	N	N	N	N	N	N	N	N
I-ASHA-FACS Muò et al. (2015)	1	N	N	N	N	N	N	N	N	N	N	N	N	N
IMD-ADL deficit Cucinotta et al. (1995)	1	N	N	N	N	N	N	N	N	N	N	N	N	N
IMD-Dem Cucinotta et al. (1995)	1	N	N	N	N	N	N	N	N	N	N	N	N	N
IPG-DepressionS Spagnoli et al. [57]	1	N	Y	Y	Y	Y	N	N	Y	Y	Y	N	N	N
ISI Castronovo et al. (2016)	1	N	N	N	N	N	N	N	N	Y	Y	N	N	N
It-RMBPC Ottoboni et al. (2019)	1	N	N	N	N	N	N	N	N	N	Y	N	N	N
LIBRA*-*index Franchini et al. (2019)	1	N	N	N	N	N	N	N	N	N	N	N	N	N
MBI-C Elefante et al. (2019)	1	N	N	N	N	N	N	N	N	N	Y	N	N	N
MDHI Sansone et al. (2017)	1	N	N	N	N	N	N	N	N	N	Y	N	N	N
MDQ Perini et al. (2016)	1	N	N/A	N/A	N/A	N/A	N/A	N/A	N/A	N/A	N	N	N	N
MDS-HC Landi et al. [25]	1	N	N	N	N	N	N	N	N	N	N	N	N	N
MOS-HIV Tozzi et al. [59]	1	N	N/A	N/A	N/A	N/A	N/A	N/A	N/A	N/A	N*	N	N	N*
MPAI-4 Cattelani et al. (2009)	1	N	N	N	N	N	N	N	N	N	Y	N	N	N
MSNQ Migliore et al. (2021)	1	N	Y	Y	N	N	N	N	Y	N	Y	N	N	N
MSQOL-29 Rosato et al. (2015)	1	Y	N/A	N/A	N/A	N/A	N/A	N/A	N/A	N/A	Y	Y	N	Y
MSQOL-54 Solari et al. [56]	1	Y	N/A	N/A	N/A	N/A	N/A	N/A	N/A	N/A	Y	Y	Y	N
Multidimensional Assessment Provinciali et al. (1999)	1	N	N	N	N	N	N	N	N	N	Y	N	Y	N
NDDI-E Mula et al. (2012)	1	N	Y	Y	Y	Y	N	N	Y	Y	Y	N	N	N
NPI-NH Baranzini et al. [3]	1	N	N	N	N	N	N	N	N	N	N*	N	N	N
NMSQuest Cova et al. (2017)	1	N	N	N	N	N	N	N	N	N	Y	N	Y	Y
NMSS Cova et al. [9]	1	N	N	N	N	N	N	N	N	N	Y	N	N	Y
NOPPAIN Ferrari et al. [15]	1	N	N/A	N/A	N/A	N/A	N/A	N/A	N/A	N/A	Y	N	Y	
NPI De Carolis et al. (2015)	1	N	N	N	N	N	N	N	N	N*	N	N	N	N
OR-PAS Santangelo et al. [53]	1	N	Y	Y	Y	Y	N	N	Y	Y	Y	Y	N	Y
PAIC Kunz et al*.* (2021)	1	Y	N/A	N/A	N/A	N/A	N/A	N/A	N/A	N/A	N	N	N	N
PAINAD Costardi et al. [8],Mosele et al. [32]	2	N	N/A	N/A	N/A	N/A	N/A	N/A	N/A	N/A	Y	N	Y	N
PASS Petrillo et al. (2021)	1	N	N	N	N	N	N	N	N	N	Y	N	Y	N
PDSS-2 Arnaldi et al. (2016)	1	N	N	N	N	N	N	N	N	Y	N	N	N	N
PRIMUS McKenna et al. (2010)	1	N	N/A	N/A	N/A	N/A	N/A	N/A	N/A	N/A	Y	N	N	N
PSDRS Quaranta et al. [43]	1	N	Y	Y	Y	Y	N	N	Y	Y	N	N	N	N
PSP-QoL Picillo et al. [41]	1	N	N/A	N/A	N/A	N/A	N/A	N/A	N/A	N/A	Y	Y	N	Y
QLQA Spaccavento et al. (2014)	1	N	N/A	N/A	N/A	N/A	N/A	N/A	N/A	N/A	N	N	N	N
QoL-AD [5]	1	N	N/A	N/A	N/A	N/A	N/A	N/A	N/A	N/A	N	N	Y	N
QOL-DyS Piacentini et al. [39]	1	Y	N/A	N/A	N/A	N/A	N/A	N/A	N/A	N/A	Y	N	N	N
QOLIBRI Giustini et al. [19],Formisano et al. [16]	2	N	N/A	N/A	N/A	N/A	N/A	N/A	N/A	N/A	Y	N	N	Y
QOLIE-31 Cramer et al. (1998); Beghi et al. (2005)	2	N	N/A	N/A	N/A	N/A	N/A	N/A	N/A	N/A	N*	N	N	Y
QSMDPD Pacchetti et al. (2004)	1	N	N/A	N/A	N/A	N/A	N/A	N/A	N/A	N/A	N	Y	N	N
SADBD*-*STROKE Monaco et al. (2005)	1	N	N	N	N	N	N	N	N	N	Y	N	N	N
SAS-6 Garofalo et al. [18]	1	N	N	N	N	N	N	N	N	Y	Y	N	N	N
SAS-I Garofalo et al. [18]	1	N	Y	Y		N	N	N	N	Y	Y	N	N	N
SDRS Lozupone et al. (2018)	1	N	Y	Y	Y	Y	N	N	Y	Y		N	N	N
SHAPS Martino et al. (2018); Santangelo et al. (2009)	2	N	N	N	N	N	N	N	N	Y	Y	Y	N	Y
SIB*-*Social Interaction Subscale Pippi et al. (1999)	1	N	N	N	N	N	N	N	N	N	N	N	N	N
SIS 3.0 Vellone et al. [61]	1	N	N/A	N/A	N/A	N/A	N/A	N/A	N/A	N/A	Y	Y	N	Y
Sleep questionnaire Manni et al. (2013)	1	N	N	N	N	N	N	N	N	N	N	N	N	N
SLOF Montemagni et al. (2015)	1	N	N	N	N	N	N	N	N	N	Y	N	N	N
STAI Santangelo et al. [53] Siciliano et al. [55],Ilardi et al. [24]	3	N	Y	Y	N	N	N	N	Y	Y	N	Y	N	Y
STAI-S Annunziata et al. (2016)	1	N	N	N	N	N	N	N	N	N	N	N	N	N
STICSA Balsamo et al. (2015)	1	N	N	N	N	N	N	N	N	N	Y	N	N	N

*Y*, explicitly investigated within reference studies; N*, not investigated within reference studies but retrievable from different ones; *N*, not investigated; *N/A*, not applicable for this tool; *AADS-I*, Assessment for Adults with Developmental Disabilities-Italian version; *ADI-12*, ALS Depression Inventory-12; *AES-C*, Apathy Evaluation Scale-Clinician version; *AES-I*, Apathy Evaluation Scale-Italian version; *AES-S*, Apathy Evaluation Scale-Self report; *AFAST*, Alzheimer’s Functional Assessment Tool; *ALS-CBSbi*, ALS Cognitive Behavioural Screen-Behavioural Inventory; *A-ONE*, Activities of daily living-focused Occupation-based Neurobehavioural Evaluation; Aphasia; *AQ-D*, Anosognosia Questionnaire-Dementia; *AUC*, Area Under the Curve; *BANS-s*, Bedford Alzheimer Nursing Severity scale; *BBI*, Beaumont Behavioural Inventory; *BDI-II*, Beck Depression Inventory-II; *BFI*, Behavioural/Functional Instrument; *BIS-15*, Barratt Impulsiveness Scale; *BPSD-DS*, Behavioural and Psychological Symptoms of Dementia-Down Syndrome; *BRIAN*, Biological Rhythms Interview of Assessment in Neuropsychiatry; *CADL-2*, Communication Activities of the Daily Living-II; *CAMDEX*, Cambridge Examination for Mental Disorders of the Elderly; *CAMDEX-I*, Cambridge Examination for Mental Disorders of the Elderly-Interview; *CarerQol*, Care-Related Quality of Life; *CBA*, Cognitive Behavioural Assessment; *CES-D*, Center for Epidemiological Studies Depression scale; *CIRS*, Clinical Insight Rating Scale; *CMT-HI*, Charcot-Marie-Tooth Health Index; *CNS*, Central nervous System; *DAD-I*, Disability Assessment Dementia scale-Italian version; *DAFS*, Direct Assessment of Functional Status; *DAS*, Dimensional Apathy Scale; *DCPR-R*, Diagnostic Criteria for Psychosomatic Research-Revised; *DHI-sf*, Dizziness Handicap Inventory-short form; *DIDA-Q*, Dual-task Impact on Daily-living Activities Questionnaire; *DS*, down syndrome; *Dys*., dysarthria; *DSM-V*, Diagnostic and Statistical Manual of mental disorders-Fifth Edition; *ELQ*, Emotional Lability Questionnaire; *eMSQOL-29-MS*, Electronic format of Multiple Sclerosis Quality of Life-29; *Epi-QoL*, Epilepsy-Quality of Life; *ESS*, Epworth Sleepiness Scale; *FAM*, Functional Assessment Measure; *FAS*, Fibromyalgia Assessment Status; *FBI-AD*, Frontal Behavioural Inventory-Alzheimer Disease; *FIM*, Functional Independence Measure; *FIQ*, Fibromyalgia Impact Questionnaire; *FLSA*, Functional Living Skills Assessment; *FTD*, Frontotemporal Dementia; *GAI*, Geriatric Anxiety Inventory; *GAS*, Geriatric Anxiety Scale; *GDS*, Geriatric Depression Scale; *GDS-15*, Geriatric Depression Scale-15; *GHS*, Geriatric Handicap Scale; *HADS*, Hospital Anxiety and Depression Scale; *HDRS*, Hamilton Depression Rating Scale; *HoNOS-RF*, Health of the Nation Outcome Scales-Residential Facilities; *I-ASHA-FACS*, Italian version of the American Speech-Language and Hearing Association-Functional Assessment of Communication Skills for adults; *IMD-ADLdeficit*, Impairment of Mental Decline-Activities of Daily Living deficit; *IMD-Dem*, Impairment of Mental Decline-Dementia; *IPG-DepressionS*, Intervista Psicogeriatrica-Depression Scales; *IRT*, Item Response Theory; *ISI*, Insomnia Severity Index; *It-RMBPC*, Italian version of the Revised Memory and Behaviour Problems Checklist; *LIBRA-index-MCI*, Lifestyle for Brain Health index-Mild Cognitive Impairment; *LR-*, Negative Likelihood Ratio; *LR* + , Positive Likelihood Ratio; *MBI*, Mild Behavioural Impairment; *MBI-C*, Mild Behavioural Impairment Checklist; *MDHI*, Myotonic Dystrophy Health Index; *MDQ*, Misidentification Delusion Questionnaire; *MDS-HC*, Minimum DataSet for Home Care; *MINI*, Mini-International Neuropsychiatric Interview; *MOS-HIV*, Medical Outcomes Study-Human Immunodeficiency Virus; *MPAI-4*, Mayo-Portland Adaptability Inventory-4; *MSNQ*, Multiple Sclerosis Neuropsychological Questionnaire; *MSQOL-29*, Multiple Sclerosis Quality Of Life-29; *MSQOL-54*, Multiple Sclerosis Quality Of Life-54; *Multid*., Multidimensional BFI (including behavioural, QoL and physical outcomes); *NDDI-E*, Neurological Disorders Depression Inventory for Epilepsy; *NMSQuest*, Non-Motor Symptoms Questionnaire; *NMSS*, Non-Motor Symptoms Scale; *NOPPAIN*, Non-Communicative Patient’s Pain Assessment Instrument; *NPI*, Neuropsychiatric Inventory; *NPV*, Negative Predictive Value; *OR-PAS*, Observer-Rated version of the Parkinson Anxiety Scale; *PAIC*, Pain Assessment in Impaired Cognition; *PAINAD*, Pain Assessment in Advanced Dementia; *PASS*, Progressive Aphasia Severity Scale; *PDSS-2*, Parkinson Disease Sleep Scale 2; *PPV*, Positive Predictive Value; *PRIMUS*, Patient-Reported Indices for Multiple Sclerosis; *PSDRS*, Post-Stroke Depression Rating Scale; *PSP-QoL*, Progressive Supranuclear Palsy-Quality of Life; *QLQA*, Quality of Life Questionnaire for Aphasics; *QoL-AD*, Quality of Life in Alzheimer’s Disease; *QOL-DyS*, Quality of Life in the Dysarthric Speaker; *QOLIBRI*, Quality of Life after Brain Injury; *QOLIE-31*, Quality of Life in Epilepsy Inventory-31 dimensions; *QSMDPD*, Questionnaire on Sleep and Mental Disorders in Parkinson’s Disease; *SADBD-STROKE*, Structured Assessment of Depression in Brain Damaged individuals-stroke; *SCD*, Subjective Cognitive Decline; *SCI*, Spinal Cord Injury; *SDRS*, Social Dysfunction Rating Scale; *SHAPS*, Snaith-Hamilton Pleasure Scale; *SIB*, Severe Impairment Battery; *SIS 3.0*, Stroke Impact Scale 3.0; *SLOF*, Specific Level Of Functioning; *STAI*, State-Trait Anxiety Inventory; *STAI-S*, State-Trait Anxiety Inventory-State Anxiety Subscale; *STICSA*, State-Trait Inventory for Cognitive and Somatic Anxiety

The most represented constructs assessed by the included BFIs were behaviour/psychiatric symptoms (apathy: *N*=6; anxiety: *N*=7; depression: *N*=11; general: *N*=13; other: *N*=11), quality of life (QoL; *N*=14) and physical (activity of daily living/functional independence: *N*=16; other: *N*=10). Multidimensional BFIs (*N*=11) included behavioural, QoL and physical constructs. Forty-one BFIs were self-report, 21 were caregiver, whereas the remaining ones clinician-report.

The vast majority of studies (*N*=109) aimed at providing psychometric, diagnostic or normative data in clinical populations, of whom 16 also addressed normotypical samples. The most represented neurological conditions were those of a degenerative/disimmune etiology: multiple sclerosis (*N*=13), amyotrophic lateral sclerosis (*N*=6) and Parkinson’s disease (*N*=10). Dementia was addressed in 17 BFIs (Alzheimer’s disease: *N*=12; vascular dementia: *N*=3; frontotemporal dementia: *N*=1; Lewy Body disease: *N*=1). Acute cerebrovascular accidents and traumatic brain injury were addressed in 5 and 3 BFIs, respectively. Nonspecific geriatric populations (see the footnotes for the available details regarding the inclusion criteria^1^) were addressed in 5 BFIs, whereas mild cognitive impairment in 8. Psychiatric populations were addressed in 4 BFIs (major depressive disorder: *N*=1; schizophrenia *spectrum* disorders: *N*=1; other: *N*=2). Three BFIs specifically addressed healthy populations. Validity was investigated for 85 BFIs, mostly by convergence (*N*=58) and divergence (*N*=34). Criterion validity was assessed for 31 BFIs, whereas content validity for 3 BFIs. Ecological validity was assessed only in 3 studies. Factorial structure underlying BFIs by means of dimensionality reduction approaches was examined in 34 BFIs.

Reliability was investigated for 80 BFIs and mostly as internal consistency (*N*=64), test-retest (*N*=39) and inter-rater (*N*=25). Parallel forms were developed for one BFI only.

Item response theory (IRT) analyses were carried out for 9 BFIs only.

Among BFIs for which diagnostic properties could be computed (*N*=79), sensitivity and specificity measures were reported for 25 tools, whereas derived metrics such as predictive values and likelihood ratios for 16. With respect to norming, when applicable (*N*=79), 21 BFIs derived norms through receiver-operating characteristics (ROC) analyses, while other methods (e.g. percentiles or *z*-scores) were address to derive cut-offs in other 31 studies. Diagnostic accuracy was tested in 21 studies.

As to feasibility, back-translation was performed in 57 BFIs; the ease of use was assessed in 12 whereas ceiling/floor effects in 22. Strikingly, time of administration was explicitly reported for very few BFIs *(N*=18).

## Discussion

### Overview

The present review provides Italian clinicians and researchers with a comprehensive, up-to-date *compendium* on available BFIs along with information on their psychometrics, diagnostics and clinical usability. This work was designed not only to serve as a guide to practitioners in selecting the appropriate tool based on the clinical questions but also to researchers involved in clinical psychometrics applied to neurology and geriatrics. In the view of raising the awareness on the statistical-methodological standards that are expected to be met by such instruments, checklists herewith delivered (BFIQA) would hopefully come in handy for orienting both the development and the psychometric/diagnostic/usability study of BFIs. Indeed, at variance with the literature on diagnostic test accuracy as applied to performance-based psychometric instruments [26], such guidelines for BFIs mostly focus on psychometrics while lacking thorough sections specifically devoted to diagnostics and clinical usability [30]. Albeit each of the BFIs included in this study can undoubtedly be recognized in its peculiarities and usefulness in research and clinical contexts, as to the level of recommendations as assessed by the BFIQA, it has to be noted that 63.5% out of those referred to clinical populations (*N*=96) fell under the pre-established cut-off of 25 (i.e. half of the full range of the scale). More specifically, the following BFIs addressed to clinical populations reached a BFIQA score ≥25: ALS Depression Inventory [36], Apathy Evaluation Scale–Self Report version [44, 51], Anosognosia Questionnaire Dementia [17], Bedford Alzheimer Nursing Severity Scale [4], Beaumont Behavioural Inventory [20], Beck Depression Inventory-II [11, 64] Care-Related Quality of Life [63], Coop/Wonca [37], Disability Assessment Dementia Scale [12], Dimensional Apathy Scale [46, 53, 54], Dual-Task Impact in Daily-living Activities Questionnaire [38], Electronic format of Multiple Sclerosis Quality of Liufe-29 [48], Epilepsy-Quality of Life [40], Frontal Behavioural Inventory [2, 29], Geriatric Handicap Scale [62], Hospital Anxiety and Depression Scale [31], Hamilton Depression Rating Scale [33, 43, 45], Medical Outcomes Study-Human Immunodeficiency Virus [59], Multiple Sclerosis Quality of Life-29 [47], Multiple Sclerosis Quality of Life-54 [56], Neuropsychiatric Invenotry–Nursing Home version [3], Non-Motor Symptoms Scale for Parkinson's disease [9], Non-Communicative Patient’s Pain Assessment Instrument [15], Observer-Rated version of the Parkinson Anxiety Scale [53], Pain Assessment in Advanced Dementia [8, 32], Progressive Supranuclear Palsy–Quality of Life [41], Quality of Life in Alzheimer’s Disease [5], Quality of Life in the Dysarthric Speaker [39], Quality of Life after Brain Injury [16, 19], Stroke Impact Scale 3.0 [61] and State-Trait Anxiety Inventory [24, 53, 55]. Moreover, out of those also or exclusively referred to normative populations (*N*=22), only 4 were classified above the same cut-off (Table 1)—Beaumont Behavioural Inventory [20], Barratt Impulsiveness Scale [28], Dimensional Apathy Scale [46, 53, 54] and Starkstein Apathy Scale [18]. Although a specific, and of course empirical, methodology has been adopted for quality assessment, such findings should warn practitioners about possible statistical and methodological lacks of several available BFIs. In this respect, several issues have been highlighted as to psychometrics, diagnostics and clinical usability of BFIs.

### Psychometrics

About two-thirds of all instruments were characterized by basic validity evidence.

However, as far as validity is concerned, its assessment was often based on convergence/divergence, whereas criterion validity was only seldom examined. With this regard, it was not uncommon that criterion validity has been tested *via* correlational, instead of regression, analyses—the latter being the proper ones to test such a property. Indeed, although the two approaches are mathematically related, while correlations are non-directional techniques solely intended to determine whether variables synchronously covary, regressions allow to test whether a first variable, which is attributed the status of a predictor, is able to account for the variability of a second one, which is instead addressed as a criterion.

In this respect, also ecological validity—testable through correlational analyses and predictive models—was infrequently investigated, raising the issue whether certain BFIs effectively reflect functional outcomes in daily life. Moreover, it is striking that factorial structure was explored in 34 BFIs only, albeit such analysis appears to be fundamental, especially for questionnaires [58]. Finally, content validity was almost never addressed: although for some BFIs can be difficult to assess content validity (e.g. in multi-domain instruments), our results strongly suggest the necessity of test such parameter by collecting ratings from experts as to the goodness of the operationalization of the target construct. We encourage this practice, as this expedient would provide practitioners with useful information about the target construct.

As to reliability, about 80% of BFIs come with such data. However, it is unfortunate to note inter-rater agreement measures lacked for 44 proxy-report BFIs, which are known to be highly subjected to heterogeneity in score attribution from examiner to examiner, also considering their different backgrounds (e.g. neurologists *vs.* psychologists). In this respect, it should be also noted that assessing inter-rater reliability in self-report BFIs is possible, albeit methodologically complex—as evidenced by the fact that such a feature was almost never assessed within included self-report BFIs. This aim could be reached, for instance, by evaluating the rate of agreement between a below- *vs.* above-cut-off classification delivered by the target BFI and that yielding from another one measuring the same construct (e.g. presence *vs.* absence of apathetic features). Indeed, if one considers that a below- vs. above-cut-off classification refers to standardized clinical judgments provided by the instruments, then such a scenario could be compared, for instance, to two clinicians (i.e. raters) evaluating a given clinical sign.

Moreover, parallel forms of included BFIs were almost never provided, limiting to an extent their usage for longitudinal applications. Although the development of parallel forms appears to be more relevant to performance-based instruments, practice effects cannot be ruled out in questionnaires either, especially those that are short-lived and thus likely to be remembered by the examinee [58].

Finally, it should be noted that, outside the framework of classical test theory, IRT analyses were almost never performed, despite them possibly providing relevant insights into the interpretation of BFI scores. In fact, while looking at total scores is crucial in order to draw clinical judgments, single item-level information would help clinicians to orient themselves towards a given diagnostic hypothesis, also possibly providing relevant prognostic information—albeit at a qualitative level. In this respect, data on item discrimination, i.e. an IRT parameter quantifying how much a given item is able to discriminate between different levels of the underlying trait, and thus the extent to which it is informative, would allow examiners to address responses to such items with greater attention. For instance, within a BFI assessing dysexecutive behavioural features, an item on the development of a sweet tooth (for instance, following the onset of a neurodegenerative condition) might result as highly informative towards the diagnosis of a frontal disorder. By contrast, within the same tool, items targeting depressive symptoms might be less informative towards such a behavioural syndrome, as being common to different brain disorders.

With that said, since practitioners and clinical researchers most of the times look at the global score yielded by a given BFI, a further useful output of potential IRT analyses might be represented by the test information function, which describes the overall informativity of the BFI based on the underlying level of the target construct. For instance, a BFI aimed at measuring apathy, which reveals itself as mostly informative for individuals having higher levels of the underlying construct (*i.e.*, high levels of apathetic features), should be used with caution when assessing patients who do not display overt symptoms (and thus are unlikely to suffer from severe apathy) since possibly yielding false negative results.

### Diagnostics

It is unfortunate to note that, out of BFIs for which diagnostics could be computed and norms derived, such data lacked for about one-third of them—this rate further dropped when addressing non-intrinsic diagnostics (i.e. predictive values and likelihood ratios). This represents a major drawback as to the clinical usability of certain BFIs as tools intended to convey diagnostic information. It is undoubtable that diagnostic properties and norms should be more accurately addressed in future studies aimed at developing and standardizing BFIs. In this respect, researchers devoted to such scopes should note that diagnostic and normative investigations do not necessarily overlap. For instance, ROC analyses allow to both derive a cut-off and to provide intrinsic/post-test diagnostics, but may be used only to the latter aim. Moreover, norms can be derived through approaches other than ROC analyses, e.g. by means of *z*-based, percentile-based or regression-based techniques.

As to the derivation of cut-offs *via* ROC analyses, it should be noted that an advisable practice would be that of providing different values based on different trade-offs between sensitivity and specificity. This would not only allow clinicians to be adaptive in selecting the most suitable cut-off values based on whether they intend to favour the sensitivity or specificity of a given BFI, but also help clinical researchers identify an adequate threshold value for inclusion/exclusion purposes in research settings. Indeed, when including a given deficit as an exclusion criterion for recruitment, stricter cut-offs might be preferred by researchers as they guarantee higher specificity and hence fewer false positives.

Finally, on the notion of “disease-specificity,” it would be reasonable not to limit the application of certain disease-specific BFIs to those clinical population(s) to which they were originally addressed. For instance, questionnaires designed to assess depression in amyotrophic lateral sclerosis (ALS) by overcoming disability-related confounders [36] might as well be applied to other motor conditions (e.g. *extra*-pyramidal disorders, multiple sclerosis). Similarly, tools assessing dysexecutive-like behavioural changes in ALS [20] might come in handy for the detection of such disturbances in other neurological conditions known to affect frontal networks (e.g. Huntington’s disease). This proposal rises from the consideration of common phenotypic manifestation possibly being underpinned by different pathophysiological processes; therefore, an extended application of disease-specific BFIs should occur only when such an assumption is met. Moreover, such “off-label” adoptions would undoubtedly need studies that support the feasibility of these disease-specific BFIs in desired populations.

### Usability

Despite being widely accepted that back-translation is required when adapting a given BFI to a new (target) language, very few BFIs appeared to undergo such a procedure, and information on BFI adaptation often lacked. Such a finding is in line with the notion according to which statistical and methodological deficiencies of psychometric instruments derived especially from cross-cultural adaptation frameworks [60].

Moreover, data on possible ceiling and/or floor effects were often unreported, preventing clinicians and researchers to evaluate whether a given BFI can be deemed as suitable for a target clinical or non-clinical population. For instance, a BFI assessing behavioural disorders and putatively presenting with a relevant ceiling/floor effect might be scarcely informative if administered with the aim of detecting sub-clinical alterations. However, such an issue is of course even more relevant when dealing with BFIs addressed to clinical populations: indeed, while ceiling/floor effects might be expected in normotypical individuals if a given BFI is aimed at detecting a clearly clinical symptoms (e.g. neuropsychiatric manifestations within the dysexecutive *spectrum*), the same would not apply for clinical populations known to present with such features (e.g. patients with frontal lobe damages). In other terms, a BFI yielding ceiling/floor effects in diseased populations is likely to be poorly usable at a clinical level. It follows that the assessment of ceiling/floor effects is mostly relevant when exploring the clinical usability of BFIs.

As for the ease of use, researchers devoted to the development and psychometric/diagnostic/usability study of BFIs are encouraged to assess how difficult a questionnaire is, from the examiner’s standpoint, to be administered, scored and interpreted, as well as, from the examinee’s standpoint, to be understood and completed. The vast majority of tools included in the present review did not come with such information.

Time requirement of BFIs was also frequently find as lacking, although this information is undoubtedly needed in order to determine whether a given tool is suitable for the target setting. For instance, not all BFIs might be adequate for bedside administrations, as being relatively long and thus scarcely appropriate to time-restricted settings. Similarly, time requirements could be different depending on whether it is in in-patient *vs.* out-patient setting.

### Further suggestions for researchers

A number of further elements, not explicitly addressed earlier in this work, can be herewith listed in order to help researchers devoted to BFI development and psychometric/diagnostic/usability study.

First, IRT analyses can be also useful, within the development of either a novel BFI or a shortened version of a previous one, to select items that adequately measure the target construct [14]. To such aims, IRT can be also complemented with classical test theory approaches in order to identify, through an empirical, data-driven approach, a set of criteria to be met in order for an item to be included into a given BFI in development—as recently proposed within the Italian scenario [28].

Second, the a priori estimation of the adequate sample size for the main target analyses within a psychometric/diagnostic/usability study for a given BFI is advisable. In this respect, a number of studies are available that suggest optimal, either empirical or simulation-based sample size estimation procedures for, e.g. validity and reliability analyses [23], dimensionality-reduction techniques [22], ROC analyses [35], IRT analyses [50] and regression-based norming [49]. In this respect, an a posteriori evaluation of the robustness of normative data can be also performed, as suggested by Crawford and Garthwaite [10].

Finally, researchers focused on BFI development and psychometric/diagnostic/usability study have to be aware of procedures aimed at handling missing data according to their categorization (e.g. at-random *vs.* not-at-random missing values) [34]. This is particularly relevant when administering several tools within a same data collection session, especially to patients: indeed, participants might not agree or be able to complete the full range of instruments included in a study protocol, e.g. due to fatigue.

## Conclusions

With the present work, practitioners have been provided with an up-to-date *compendium* of available BFIs in Italy and also to present some possible criticisms about their properties, and deliver hopefully useful insights into best-practice guidelines. To this last aim, it is believed that the BFIQA scales herewith provided may serve as a plot for researchers in order to carefully consider relevant aspects associated with the development and psychometric/diagnostic/usability of BFIs, in order to strengthen their level of recommendation for their use in clinical practice as applied to diagnostic, prognostic and interventional setting.

## Supplementary Information

Below is the link to the electronic supplementary material.

Supplementary file1 (DOCX 30 kb)

Supplementary file2 (DOCX 33 kb)

## Data Availability

Data collected and analyzed within the present study are accessible upon reasonable request to the Corresponding Author.

## Footnotes

1 NPI-NH [3]: patients with a diagnosis of dementia and/or psychiatric disorders, according to DSM-IV-TR; AES-I [7]: patients with either major depressive disorder or dementia and community-dwelling controls with a Mini-Mental State Examination raw score > 18; MDS-HC [25]: all eligible individuals that could benefit from home care services; IPGDepressionS [57]: residents in geriatric institutions; GHS [62]: community-dwelling elderlies.
