# Supplementary material for: Psychometrics, diagnostics and usability of Italian tools assessing behavioural and functional outcomes in neurological, geriatric and psychiatric disorders: a systematic review
Source: Neurol Sci. 2022 Aug 6;43(11):6189–214. doi: 10.1007/s10072-022-06300-8 (PMC9616758; doi:10.1007/s10072-022-06300-8)
Supplement: Supplementary file 1 — Supplementary file1 (DOCX 30 kb) [file 10072_2022_6300_MOESM1_ESM.docx]

**Behavioural and Functional Instrument Quality Assessment - Normative Sample (BFIQA-NS).**

| **Sampling (BFIQA-NS-S)** | |
| --- | --- |
| **Are sample demographics reported?** | |
| Age, education and sex reported | 1 |
| ≥1 feature(s) missing (*i.e.,* age, education or sex) | 0 |
| **Are sample demographics adequately described?** | |
| Continuous ones (*e.g.*, age and education) reported as *M*±*SD* and *range*; categorical ones as frequencies/percentages (*e.g.*, sex) | 1 |
| ≥1 statistics not reported | 0 |
| **Is(are) the normative sample(s) size adequate?** | |
| *N*≥200 | 2 |
| 100≤*N*<200 | 1 |
| *N*<100 | 0 |
| **Has a power analysis carried out?** (1 if “yes”) | 1 |
| **Is(are) the normative sample(s) geographically representative?** |  |
| At least 3 different regions with acceptable geographic coverage (*e.g.,* North, Center, South) | 2 |
| At least 2 different region with acceptable geographic coverage (*e.g*., North and South) | 1 |
| One region only | 0 |
| **Does(do) the normative sample(s) cover a sufficiently wide range of adult age?** | |
| *Range*≥50 | 2 |
| *3*0≤*range*<50 | 1 |
| *Range*<30 | 0 |
| **Is(are) the normative sample(s) representative of all levels of education?** | |
| *Range*≥13 | 2 |
| *1*0≤*range*< 13 | 1 |
| *Range*<10 | 0 |
| **Is(are) the normative sample(s) stratification adequate (for age, education and sex)?** | |
| The majority of cells including at least 1 observation, except for “critical” ones (*e.g*., young age with low education) | 1 |
| Clearly unbalanced patterns detectable within table stratification | 0 |
| **Is(are) the normative sample(s) well balanced between males and females** (40 and 60% or 50-50% circa, respectively)? | 1 |
| **Are exclusion criteria for normative sample(s) adequately described?** |  |
| Quantitative (*e.g.,* cut-off scores on a test) & qualitative (*e.g.*, premorbid conditions) | 2 |
| Either one of each (quantitative or well-described qualitative) | 1 |
| Only qualitative and not sufficiently described | 0 |
| **BFIQA-NS-S score:** | **__/15** |
| **Psychometrics, diagnostics and usability (BFIQA-NS-PDU)** | |
| **Is linguistic adaptation adequate?** | |
| Back-translation/not necessary (*de novo* instrument) | 2 |
| Simple translation with adequate controls for subjectivity biases (*e.g*., independent judges) | 1 |
| Simple translation only | 0 |
| **Is cross-cultural adaptation adequate (if necessary)?** | |
| Critical items adequately addressed/not necessary | 1 |
| Critical items not adequately addressed | 0 |
| **Is there acceptability evidence?** (1 if “yes”) | 1 |
| **Have ceiling/floor effects been investigated?** (1 if “yes”) | 1 |
| **Has validity been tested?** (1 if “yes”) | 1 |
| **If “yes” which of the following validity measures have been considered?** (1 if “yes”) | |
| Convergent validity | 1 |
| Divergent validity | 1 |
| Criterion validity | 1 |
| Ecological validity | 1 |
| Face validity | 1 |
| Dimensionality-reduction techniques | 1 |
| Other validity measures | 1 |
| **If convergent validity has been tested: is(are) the correlational measure(s) appropriate (*i.e.,* targeting the same construct)?** (1 if “yes”) | 1 |
| **Has reliability been tested?** (1 if “yes”) | 1 |
| **If “yes”, which of the following reliability measures has been considered?** (1 if “yes”) | |
| Inter-rater reliability | 1 |
| Test-retest reliability | 1 |
| Internal consistency | 1 |
| Parallel forms/significant change measures | 1 |
| **Has an Item Response Theory analysis been carried out?** (1 if “yes”) | 1 |
| **Has sensitivity been tested?** (1 if “yes”)* | 1 |
| **Has specificity been tested?** (1 if “yes”)* | 1 |
| **Are sensitivity- and specificity-derived metrics present? (*e.g*., PPV)** (1 if “yes”)* | 1 |
| **Is accuracy (*e.g.*, AUC) reported?** (1 if “yes”)* | 1 |
| **If ROC analyses have been carried out:** | |
| Has the cut-off value been identified with an explicitly described procedure (*e.g.,* Youden index)?* | 1 |
| **Has the target condition been adequately identified?** |  |
| Quantitative (*e.g.*, cut-off scores on a test) | 1 |
| Qualitative (*e.g.*, clinical criteria) | 1 |
| **Has at least one cut-off value derived?** (1 if “yes”)* | 1 |
| **Is the ease of tool use adequately evaluated?** |  |
| Both from the examiner’s and the examinee’s standpoint | 2 |
| Either from the examiner’s or from the examinee’s standpoint | 1 |
| Neither from the examiner’s nor from the examinee’s standpoint | 0 |
| **Is information about time of use reported?** (1 if “yes”) | 1 |
| **Is this a translation + standardization study?** |  |
| Translation + standardization | 2 |
| Not applicable (experimental study) | 1 |
| Only translation | 0 |
| **Is this a single-domain or a multidimensional tool?** |  |
| Single-domain tool | 2 |
| Multidimensional tool | 1 |
| **BFIQA-NS-PDU score:** | **_/35** |
| **BFIQA-NS total score:** | **_/50** |
| **Notes.** AUC=area under the curve; ROC=receiver-operating characteristics; PPV=positive predictive value.  Unreported information were scored as 0. *For BFIs assessing quality of life and pain, items regarding diagnostics were attributed the maximum as being not applicable; items about sample representativeness were evaluated as NA if no clear epidemiological data about the target condition were available (*e.g*., Charcot-Marie-Tooth Syndrome). | |

**Behavioural and Functional Instrument Quality Assessment - Clinical Population (BFIQA-CP).**

| **Sampling (BFIQA-CP-S)** | |
| --- | --- |
| **Are sample demographics reported?** | |
| Age, education and sex reported | 1 |
| ≥1 feature(s) missing (*i.e.,* age, education or sex) | 0 |
| **Are sample demographics adequately described?** | |
| Continuous ones (*e.g.*, age and education) reported as *M*±*SD* and *range*; categorical ones as frequencies/percentages (*e.g.*, sex) | 1 |
| ≥1 statistics not reported | 0 |
| **Is(are) the clinical sample(s) size adequate?** | |
| *N*≥50 | 2 |
| 30≤*N*<50 | 1 |
| *N*<30 | 0 |
| **Are clinical features adequately described?** | |
| Continuous ones (*e.g.*, disease duration) reported as *M*±*SD* and *range*; categorical ones as frequencies/percentages (*e.g.*, treated/untreated) | 2 |
| ≥ 50% statistics not reported | 1 |
| Statistics not reported at all | 0 |
| **Does(do) the clinical sample(s) cover a sufficiently wide range of adult age?** | |
| *Range*≥30 | 2 |
| 10≤*range*<30 | 1 |
| *Range*<10 | 0 |
| **Is(are) the clinical sample(s) representative of the sex ratio of the target condition(s)** (*e.g.*, males>females in Parkinson’s disease)?* | 1 |
| **Are inclusion criteria for clinical population(s) adequately described?** |  |
| Clinical diagnoses supported by *ad hoc* criteria | 2 |
| Sufficiently detailed clinical diagnoses only | 1 |
| Insufficiently detailed clinical diagnoses | 0 |
| **Is the presence of comorbidities adequately reported?** |  |
| neurological/psychiatric and medical-general conditions | 2 |
| either neurological/psychiatric or medical-general conditions | 1 |
| neither neurological/psychiatric nor medical-general conditions | 0 |
| **Number of clinical populations the instrument has been validated in:** |  |
| ≥2 | 2 |
| 1 | 1 |
| **BFIQA-CP-S score:** | **__/15** |
| **Psychometrics, diagnostics and usability (BFIQA-CP-PDU)** | |
| **Is linguistic adaptation adequate?** | |
| Back-translation/not necessary (*de novo* instrument) | 2 |
| Simple translation with adequate controls for subjectivity biases (*e.g*., independent judges) | 1 |
| Simple translation only | 0 |
| **Is cross-cultural adaptation adequate (if necessary)?** | |
| Critical items adequately addressed/not necessary | 1 |
| Critical items not adequately addressed | 0 |
| **Is there acceptability evidence?** (1 if “yes”) | 1 |
| **Have ceiling/floor effects been investigated?** (1 if “yes”) | 1 |
| **Has validity been tested?** (1 if “yes”) | 1 |
| **If “yes” which of the following validity measures have been considered?** (1 if “yes”) | |
| Convergent validity | 1 |
| Divergent validity | 1 |
| Criterion validity | 1 |
| Ecological validity | 1 |
| Face validity | 1 |
| Dimensionality-reduction techniques | 1 |
| Other validity measures | 1 |
| **If convergent validity has been tested: is/are the correlational measure(s) appropriate (i.e., targeting the same construct)?** (1 if “yes”) | 1 |
| **Has reliability been tested?** (1 if “yes”) | 1 |
| **If “yes”, which of the following reliability measures has been considered?** (1 if “yes”) | |
| Inter-rater reliability | 1 |
| Test-retest reliability | 1 |
| Internal consistency | 1 |
| Parallel forms/significant change measures | 1 |
| **Has an Item Response Theory analysis been carried out?** (1 if “yes”) | 1 |
| **Has sensitivity been tested?** (1 if “yes”)* | 1 |
| **Has specificity been tested?** (1 if “yes”)* | 1 |
| **Are sensitivity- and specificity-derived metrics present? (*e.g*., PPV)** (1 if “yes”)* | 1 |
| **Is accuracy (*e.g.*, AUC) reported?*** | 1 |
| **If ROC analyses have been carried out** (1 if “yes”)**:** | |
| Has the cut-off value been identified with an explicitly described procedure (*e.g.,* Youden index)? | 1 |
| **Has the target condition been adequately identified?** |  |
| Quantitative (*e.g.*, cut-off scores on a test) | 1 |
| Qualitative (*e.g.*, clinical criteria) | 1 |
| **Has at least one cut-off value derived?** (1 if “yes”)* | 1 |
| **Is the ease of tool use adequately evaluated?** |  |
| Both from the examiner’s and the examinee’s standpoint | 2 |
| Either from the examiner’s or from the examinee’s standpoint | 1 |
| Neither from the examiner’s nor from the examinee’s standpoint | 0 |
| **Is information about time of use reported?** (1 if “yes”) | 1 |
| **Is this a translation + standardization study?** |  |
| Translation + standardization | 2 |
| Not applicable (*e.g.,* experimental study, only validation study) | 1 |
| Only translation | 0 |
| **Is this a single-domain or a multidimensional tool?** |  |
| Single-domain tool | 2 |
| Multidimensional tool | 1 |
| **BFIQA-CP-PDU** | **_/35** |
| **BFIQA-CP total score:** | **_/50** |
| **Notes.** AUC=area under the curve; ROC=receiver-operating characteristics; PPV=positive predictive value.  Unreported information were scored as 0. *For BFIs assessing quality of life and pain, items regarding diagnostics were attributed the maximum as being not applicable; items about sample representativeness were evaluated as NA if no clear epidemiological data about the target condition were available (*e.g*., Charcot-Marie-Tooth Syndrome). | |
