# Supplementary material for: Psychometrics, diagnostics and usability of Italian tools assessing behavioural and functional outcomes in neurological, geriatric and psychiatric disorders: a systematic review
Source: Neurol Sci. 2022 Aug 6;43(11):6189–214. doi: 10.1007/s10072-022-06300-8 (PMC9616758; doi:10.1007/s10072-022-06300-8)
Supplement: Supplementary file 2 — Supplementary file2 (DOCX 33 kb) [file 10072_2022_6300_MOESM2_ESM.docx]

**Appendix 1.** List of references for the included studies.

1. Alberici A, Geroldi C, Cotelli M, et al (2007) The Frontal Behavioural Inventory (Italian version) differentiates frontotemporal lobar degeneration variants from Alzheimer’s disease. Neurol Sci 28:80–86. https://doi.org/10.1007/s10072-007-0791-3
2. Annunziata MA, Muzzatti B, Flaiban C, et al (2018) Cognitive Functioning Self-Assessment Scale (CFSS): Further psychometric data. Appl Neuropsychol. https://doi.org/10.1080/23279095.2016.1225575
3. Arnaldi D, Cordano C, De Carli F, et al (2016) Parkinson’s Disease Sleep Scale 2: application in an Italian population. Neurol Sci. https://doi.org/10.1007/s10072-015-2409-5
4. Balsamo M, Innamorati M, Van Dam NT, et al (2015) Measuring anxiety in the elderly: Psychometric properties of the state trait inventory of cognitive and somatic anxiety (STICSA) in an elderly Italian sample. Int Psychogeriatrics 27:999–1008. https://doi.org/10.1017/S1041610214002634
5. Baranzini F, Grecchi A, Berto E, et al (2013) Factor analysis and psychometric properties of the Italian version of the Neuropsychiatric Inventory-Nursing Home in a population of institutionalized elderly in psychiatric comorbidity. Riv. Psichiatr. 48:335–344
6. Beghi E, Niero M, Roncolato M (2005) Validity and reliability of the Italian version of the Quality-of-Life in Epilepsy Inventory (QOLIE-31). Seizure 14:452–458. https://doi.org/10.1016/j.seizure.2005.07.008
7. Bellelli G, Frisoni GB, Bianchetti A, Trabucchi M (1997) The Bedford Alzheimer Nursing Severity scale for the severely demented: Validation study. Alzheimer Dis Assoc Disord 11:71–77. https://doi.org/10.1097/00002093-199706000-00003
8. Bertolotti G, Michielin P, Vidotto G, et al (2015) Metric qualities of the cognitive behavioral assessment for outcome evaluation to estimate psychological treatment effects. Neuropsychiatr Dis Treat 11:2449–2460. https://doi.org/10.2147/NDT.S86855
9. Bianchetti A, Cornali C, Ranieri P, Trabucchi M (2017) Quality of life in patients with mild dementia. Validation of the Italian version of the quality of life Alzheimer’s disease (QoL-AD) scale. J Gerontol Geriatr 65:137–143
10. Borgi M, Caccamo F, Giuliani A, et al (2016) Validation of the Italian version of the Apathy Evaluation Scale (AES-I) in institutionalized geriatric patients. Ann Ist Super Sanita 52:249–255. https://doi.org/10.4415/ANN_16_02_17
11. Caracciolo B, Giaquinto S (2002) Criterion validity of the Center for Epidemiological Studies Depression (CES-D) scale in a sample of rehabilitation inpatients. J Rehabil Med 34:221–225. https://doi.org/10.1080/165019702760279215
12. Castiglia SF, Galeoto G, Lauta A, et al (2017) The culturally adapted Italian version of the Barthel Index (IcaBI): Assessment of structural validity, inter-rater reliability and responsiveness to clinically relevant improvements in patients admitted to inpatient rehabilitation centers. Funct Neurol 32:221–228. https://doi.org/10.11138/FNeur/2017.32.4.221
13. Castronovo V, Galbiati A, Marelli S, et al (2016) Validation study of the Italian version of the Insomnia Severity Index (ISI). Neurol Sci 37:1517–1524. https://doi.org/10.1007/s10072-016-2620-z
14. Cattelani R, Corsini D, Posteraro L, et al (2009) The Italian version of the Mayo-Portland adaptability inventory-4. A new measure of brain injury outcome. Eur J Phys Rehabil Med
15. Cerone M, Tofani M, Fabbrini G, et al (2021) Evaluation of the Psychometric Properties of the Scale A-ONE: An Italian Cross-Sectional Study. Occup Ther Int 2021:. https://doi.org/10.1155/2021/8874953
16. Chiesi F, Primi C, Pigliautile M, et al (2018) Does the 15-item Geriatric Depression Scale function differently in old people with different levels of cognitive functioning? J Affect Disord 227:471–476. https://doi.org/10.1016/j.jad.2017.11.045
17. Cosci F, Svicher A, Romanazzo S, et al (2019) Criterion-related validity in a sample of migraine outpatients: The diagnostic criteria for psychosomatic research. CNS Spectr. https://doi.org/10.1017/S1092852919001536
18. Costardi D, Rozzini L, Costanzi C, et al (2007) The Italian version of the pain assessment in advanced dementia (PAINAD) scale. Arch Gerontol Geriatr. https://doi.org/10.1016/j.archger.2006.04.008
19. Cova I, Di Battista ME, Vanacore N, et al (2017) Validation of the Italian version of the Non Motor Symptoms Scale for Parkinson’s disease. Park Relat Disord 34:38–42. https://doi.org/10.1016/j.parkreldis.2016.10.020
20. Cova I, Di Battista ME, Vanacore N, et al (2017) Adaptation and psychometric properties of the Italian version of the Non-Motor Symptoms Questionnaire for Parkinson’s disease. Neurol Sci 38:673–678. https://doi.org/10.1007/s10072-017-2830-z
21. Cramer JA, Perrine K, Devinsky O, et al (1998) Development and cross-cultural translations of a 31-item quality of life in epilepsy inventory. Epilepsia 39:81–88. https://doi.org/10.1111/j.1528-1157.1998.tb01278.x
22. Cucinotta D, Ambrosoli L, Poli A, et al (1995) Clinical assessment of mental decline in elderly people: A proposal for a new quantitative index. Aging Clin Exp Res 7:29–34. https://doi.org/10.1007/BF03324289
23. Cuoco S, Cappiello A, Abate F, et al (2021) Psychometric properties of the Beck Depression Inventory-II in progressive supranuclear palsy. Brain Behav 11:. https://doi.org/10.1002/brb3.2344
24. De Carolis A, Cipollini V, Corigliano V, et al (2015) Anosognosia in people with cognitive impairment: Association with cognitive deficits and behavioral disturbances. Dement Geriatr Cogn Dis Extra 5:42–50. https://doi.org/10.1159/000367987
25. De Vreese LP, Mantesso U, De Bastiani E, et al (2011) Psychometric evaluation of the Italian version of the AADS questionnaire: A caregiver-rated tool for the assessment of behavioral deficits and excesses in persons with intellectual disabilities and dementia. Int Psychogeriatrics 23:1124–1132. https://doi.org/10.1017/S1041610211000342
26. De Vreese LP, Gomiero T, Uberti M, et al (2015) Functional abilities and cognitive decline in adult and aging intellectual disabilities. Psychometric validation of an Italian version of the Alzheimer’s Functional Assessment Tool (AFAST): Analysis of its clinical significance with linear statistics and artificial neural networks. J Intellect Disabil Res 59:370–384. https://doi.org/10.1111/jir.12113
27. De Vreese LP, Caffarra P, Savarè R, et al (2008) Functional disability in early Alzheimer’s disease - A validation study of the Italian version of the Disability Assessment for Dementia scale. Dement Geriatr Cogn Disord 25:186–194. https://doi.org/10.1159/000113415
28. Dekker AD, Sacco S, Carfi A, et al (2018) The Behavioral and Psychological Symptoms of Dementia in Down Syndrome (BPSD-DS) Scale: Comprehensive Assessment of Psychopathology in Down Syndrome. J Alzheimer’s Dis 63:797–820. https://doi.org/10.3233/JAD-170920
29. Elefante C, Lattanzi L, Ismail Z, et al (2019) Mild behavioral impairment: Presentation of the diagnostic criteria and the Italian version of the MBI-Checklist. Riv Psichiatr 54:59–66. https://doi.org/10.1708/3142.31246
30. Farina E, Fioravanti R, Pignatti R, et al (2010) Functional living skills assessment: A standardized measure of high-order activities of daily living in patients with dementia. Eur J Phys Rehabil Med 46:73–80
31. Ferrari R, Martini M, Mondini S, et al (2009) Pain assessment in non-communicative patients: The Italian version of the Non-Communicative Patient’s Pain Assessment Instrument (NOPPAIN). Aging Clin Exp Res 21:298–306. https://doi.org/10.1007/BF03324919
32. Formisano R, Longo E, Azicnuda E, et al (2017) Quality of life in persons after traumatic brain injury as self-perceived and as perceived by the caregivers. Neurol Sci 38:279–286. https://doi.org/10.1007/s10072-016-2755-y
33. Franchignoni FP, Benevolo E, Zelaschi GP, et al (1995) La Scala Fim Nel Monitoraggio Del Recupero Funzionale Neuromotorio: Studio Su Emiplegici in Degenza Riabilitativa Post-Acuta. Eura Medicophys 31:67–75
34. Franchini F, Musicco M, Ratto F, et al (2019) The LIBRA Index in Relation to Cognitive Function, Functional Independence, and Psycho-Behavioral Symptoms in a Sample of Non-Institutionalized Seniors at Risk of Dementia. J Alzheimer’s Dis 72:717–731. https://doi.org/10.3233/JAD-190495
35. Furneri G, Platania S, Privitera A, et al (2021) The apathy evaluation scale (Aes-c): Psychometric properties and invariance of italian version in mild cognitive impairment and alzheimer’s disease. Int J Environ Res Public Health 18:. https://doi.org/10.3390/ijerph18189597
36. Galeoto G, Sansoni J, Scuccimarri M, et al (2018) A Psychometric Properties Evaluation of the Italian Version of the Geriatric Depression Scale. Depress Res Treat 2018:. https://doi.org/10.1155/2018/1797536
37. Gambina G, Valbusa V, Corsi N, et al (2015) The Italian validation of the anosognosia questionnaire for dementia in Alzheimer’s disease. Am J Alzheimers Dis Other Demen 30:635–644. https://doi.org/10.1177/1533317515577185
38. Garofalo E, Iavarone A, Chieffi S, et al (2021) Italian version of the Starkstein Apathy Scale (SAS-I) and a shortened version (SAS-6) to assess “pure apathy” symptoms: normative study on 392 individuals. Neurol Sci 42:1065–1072. https://doi.org/10.1007/s10072-020-04631-y
39. Giustini M, Longo E, Azicnuda E, et al (2014) Health-related quality of life after traumatic brain injury: Italian validation of the qolibri. Funct Neurol 29:167–176. https://doi.org/10.11138/FNeur/2014.29.3.167
40. Iannuccelli C, Sarzi-Puttini P, Atzeni F, et al (2011) Psychometric properties of the Fibromyalgia Assessment Status (FAS) index: A national web-based study of fibromyalgia. Clin Exp Rheumatol 29:
41. Iazzolino B, Pain D, Laura P, et al (2022) Italian adaptation of the Beaumont Behavioral Inventory (BBI): psychometric properties and clinical usability. Amyotroph Lateral Scler Front Degener 23:81–86. https://doi.org/10.1080/21678421.2021.1946085
42. Ilardi CR, Gamboz N, Iavarone A, et al (2021) Psychometric properties of the STAI-Y scales and normative data in an Italian elderly population. Aging Clin Exp Res 33:2759–2766. https://doi.org/10.1007/s40520-021-01815-0
43. Kunz M, Crutzen-Braaksma P, Giménez-Llort L, et al (2021) Observing pain in individuals with cognitive impairment: A pilot comparison attempt across countries and across different types of cognitive impairment. Brain Sci. https://doi.org/10.3390/brainsci11111455
44. Landi F, Tua E, Onder G, et al (2000) Minimum data set for home care: A valid instrument to assess frail older people living in the community. Med Care 38:1184–1190. https://doi.org/10.1097/00005650-200012000-00005
45. Lawton G, Lundgren-Nilsson Å, Biering-Sørensen F, et al (2006) Cross-cultural validity of FIM in spinal cord injury. Spinal Cord 44:746–752. https://doi.org/10.1038/sj.sc.3101895
46. Lozupone M, Panza F, Piccininni M, et al (2018) Social dysfunction in older age and relationships with cognition, depression, and apathy: The GreatAGE Study. J Alzheimer’s Dis 65:989–1000. https://doi.org/10.3233/JAD-180466
47. Lundgren-Nilsson Å, Grimby G, Ring H, et al (2005) Cross-cultural validity of Functional Independence Measure items in stroke: A study using Rasch analysis. J Rehabil Med 37:23–31. https://doi.org/10.1080/16501970410032696
48. Maggi G, Altieri M, Ilardi CR, Santangelo G (2022) Validation of a short Italian version of the Barratt Impulsiveness Scale (BIS-15) in non-clinical subjects: psychometric properties and normative data. Neurol Sci. https://doi.org/10.1007/s10072-022-06047-2
49. Manni R, Sinforiani E, Zucchella C, et al (2013) A sleep continuity scale in Alzheimer’s disease: Validation and relationship with cognitive and functional deterioration. Neurol Sci 34:701–705. https://doi.org/10.1007/s10072-012-1118-6
50. Martinez-Martin P, Radicati FG, Rodriguez Blazquez C, et al (2019) Extensive validation study of the Parkinson’s Disease Composite Scale. Eur J Neurol 26:1281–1288. https://doi.org/10.1111/ene.13976
51. Martino I, Santangelo G, Moschella D, et al (2018) Assessment of Snaith-Hamilton Pleasure Scale (SHAPS): the dimension of anhedonia in Italian healthy sample. Neurol Sci 39:657–661. https://doi.org/10.1007/s10072-018-3260-2
52. McKenna SP, Doward LC, Twiss J, et al (2010) International development of the patient-reported outcome indices for multiple sclerosis (PRIMUS). Value Heal 13:946–951. https://doi.org/10.1111/j.1524-4733.2010.00767.x
53. Migliore S, Landi D, Proietti F, et al (2021) Validity of the Italian multiple sclerosis neuropsychological screening questionnaire. Neurol Sci 42:4583–4589. https://doi.org/10.1007/s10072-021-05141-1
54. Milan G, Lamenza F, Iavarone A, et al (2008) Frontal Behavioural Inventory in the differential diagnosis of dementia. Acta Neurol Scand 117:260–265. https://doi.org/10.1111/j.1600-0404.2007.00934.x
55. Monaco F, Mazzini L, Marchetti C, et al (2005) The structured assessment of depression in brain-damaged individuals: Translation and validation study of the Italian version. Neurol Sci 26:182–184. https://doi.org/10.1007/s10072-005-0459-9
56. Mondolo F, Jahanshahi M, Granà A, et al (2006) The validity of the hospital anxiety and depression scale and the geriatric depression scale in Parkinson’s disease. Behav Neurol 17:109–115. https://doi.org/10.1155/2006/136945
57. Montemagni C, Rocca P, Mucci A, et al (2015) Italian version of the “Specific Level of Functioning.” J Psychopathol 21:287–296
58. Moro MF, Carta MG, Pintus M, et al (2014) Validation of the Italian Version of the Biological Rhythms Interview of Assessment in Neuropsychiatry (BRIAN): Some Considerations on its Screening Usefulness. Clin Pract Epidemiol Ment Heal 10:48–52. https://doi.org/10.2174/1745017901410010048
59. Mosele M, Inelmen EM, Toffanello ED, et al (2012) Psychometric properties of the pain assessment in advanced dementia scale compared to self assessment of pain in elderly patients. Dement Geriatr Cogn Disord. https://doi.org/10.1159/000341582
60. Mula M, Iudice A, La Neve A, et al (2012) Validation of the Italian version of the Neurological Disorders Depression Inventory for Epilepsy (NDDI-E). Epilepsy Behav 24:329–331. https://doi.org/10.1016/j.yebeh.2012.04.130
61. Mula M, Iudice A, La Neve A, et al (2014) Validation of the Hamilton Rating Scale for Depression in adults with epilepsy. Epilepsy Behav 41:122–125. https://doi.org/10.1016/j.yebeh.2014.08.029
62. Muò R, Cancialosi P, Galimberti L, et al (2015) Validation of the Italian version of the American Speech-Language and Hearing Association—Functional Assessment of Communication Skills for adults (I-ASHA-FACS). Aphasiology 29:1110–1130. https://doi.org/10.1080/02687038.2015.1010475
63. Neri M, Roth M, Rubichi S, et al (2001) The validity of informant report for grading the severity of Alzheimer’s dementia. Aging Clin Exp Res 13:22–29. https://doi.org/10.1007/bf03351490
64. Neri M, Andermarcher E, Spanó A, et al (1992) Validation study of the italian version of the cambridge mental disorders of the elderly examination: Preliminary findings. Dement Geriatr Cogn Disord 3:70–77. https://doi.org/10.1159/000106997
65. Ottoboni G, Amici S, Iannizzi P, et al (2019) Italian revised memory and behavior problems checklist (It-RMBPC): validation and psychometric properties in Alzheimer’s disease caregivers. Aging Clin Exp Res 31:527–537. https://doi.org/10.1007/s40520-018-0995-9
66. Pacchetti C, Manni R, Zangaglia R, et al (2004) A questionnaire on sleep and mental disorders in Parkinson’s disease (QSMDPD): Development and application of a new screening tool. Funct Neurol 19:83–99
67. Pain D, Aiello EN, Gallucci M, et al (2021) The Italian Version of the ALS Depression Inventory-12. Front Neurol 12:. https://doi.org/10.3389/fneur.2021.723776
68. Palmieri A, Abrahams S, Sorarù G, et al (2009) Emotional Lability in MND: Relationship to cognition and psychopathology and impact on caregivers. J Neurol Sci 278:16–20. https://doi.org/10.1016/j.jns.2008.10.025
69. Pappalardo A, Chisari CG, Montanari E, et al (2017) The clinical value of Coop/Wonca charts in assessment of HRQoL in a large cohort of relapsing-remitting multiple sclerosis patients: Results of a multicenter study. Mult Scler Relat Disord 17:154–171. https://doi.org/10.1016/j.msard.2017.07.022
70. Pedrini L, Lanfredi M, Ferrari C, et al (2018) Development and Validation of the Health of the Nation Outcome Scales-Residential Facility (HoNOS-RF). Psychiatr Q 89:461–473. https://doi.org/10.1007/s11126-017-9548-1
71. Pedullà L, Tacchino A, Podda J, et al (2020) The patients’ perspective on the perceived difficulties of dual-tasking: development and validation of the Dual-task Impact on Daily-living Activities Questionnaire (DIDA-Q). Mult Scler Relat Disord 46:. https://doi.org/10.1016/j.msard.2020.102601
72. Perini G, Carlini A, Pomati S, et al (2016) Misidentification Delusions: Prevalence in Different Types of Dementia and Validation of a Structured Questionnaire. Alzheimer Dis Assoc Disord 30:331–337. https://doi.org/10.1097/WAD.0000000000000141
73. Petrillo SM, Del Mauro M, Lambro BE, et al (2021) Italian translation and cross-cultural adaptation of the Progressive Aphasia Severity Scale. Neurol Sci. https://doi.org/10.1007/s10072-021-05651-y
74. Piacentini V, Zuin A, Cattaneo D, Schindler A (2011) Reliability and validity of an instrument to measure quality of life in the dysarthric speaker. Folia Phoniatr Logop 63:289–295. https://doi.org/10.1159/000322800
75. Piazzini A, Beghi E, Turner K, et al (2008) Health-related quality of life in epilepsy: Findings obtained with a new Italian instrument. Epilepsy Behav 13:119–126. https://doi.org/10.1016/j.yebeh.2008.02.017
76. Picconi L, Balsamo M, Palumbo R, Fairfield B (2018) Testing factor structure and measurement invariance across gender with Italian Geriatric Anxiety Scale. Front Psychol 9:. https://doi.org/10.3389/fpsyg.2018.01164
77. Picillo M, Cuoco S, Amboni M, et al (2019) Validation of the Italian version of the PSP Quality of Life questionnaire. Neurol Sci 40:2587–2594. https://doi.org/10.1007/s10072-019-04010-2
78. Pickering G, Gibson SJ, Serbouti S, et al (2010) Reliability study in five languages of the translation of the pain behavioural scale Doloplus®. Eur J Pain 14:545.e1-545.e10. https://doi.org/10.1016/j.ejpain.2009.08.004
79. Pigliautile M, Chiesi F, Primi C, et al (2019) Validation study of the Italian version of Communication Activities of the Daily Living (CADL 2) as an ecologic cognitive assessment measure in older subjects. Neurol Sci 40:2081–2088. https://doi.org/10.1007/s10072-019-03937-w
80. Pippi M, Mecocci P, Saxton J, et al (1999) Neuropsychological assessment of the severely impaired elderly patient: Validation of the Italian short version of the Severe Impairment Battery (SIB). Aging Clin Exp Res 11:221–226. https://doi.org/10.1007/bf03339662
81. Pisciotta C, Ciafaloni E, Zuccarino R, et al (2020) Validation of the Italian version of the Charcot-Marie-Tooth Health Index. J Peripher Nerv Syst 25:292–296. https://doi.org/10.1111/jns.12397
82. Provinciali L, Ceravolo MG, Bartolini M, et al (1999) A multidimensional assessment of multiple sclerosis: Relationships between disability domains. Acta Neurol Scand 100:156–162. https://doi.org/10.1111/j.1600-0404.1999.tb00731.x
83. Quaranta D, Marra C, Gainotti G (2008) Mood disorders after stroke: Diagnostic validation of the poststroke depression rating scale. Cerebrovasc Dis 26:237–243. https://doi.org/10.1159/000147450
84. Raimo S, Trojano L, Gaita M, et al (2020) Assessing apathy in multiple sclerosis: Validation of the dimensional apathy scale and comparison with apathy evaluation scale. Mult Scler Relat Disord 38:. https://doi.org/10.1016/j.msard.2019.101870
85. Raimo S, Trojano L, Spitaleri D, et al (2014) Apathy in multiple sclerosis: A validation study of the apathy evaluation scale. J Neurol Sci 347:295–300. https://doi.org/10.1016/j.jns.2014.10.027
86. Raimo S, Trojano L, Spitaleri D, et al (2015) Psychometric properties of the Hamilton Depression Rating Scale in multiple sclerosis. Qual Life Res 24:1973–1980. https://doi.org/10.1007/s11136-015-0940-8
87. Rinaldi P, Mecocci P, Benedetti C, et al (2003) Validation of the five-item Geriatric Depression Scale in elderly subjects in three different settings. J Am Geriatr Soc 51:694–698. https://doi.org/10.1034/j.1600-0579.2003.00216.x
88. Rosato R, Testa S, Bertolotto A, et al (2016) Development of a short version of MSQOL-54 using factor analysis and item response theory. PLoS One 11. https://doi.org/10.1371/journal.pone.0153466
89. Rosato R, Testa S, Bertolotto A, et al (2019) eMSQOL-29: Prospective validation of the abbreviated, electronic version of MSQOL-54. Mult Scler J 25:856–866. https://doi.org/10.1177/1352458518774935
90. Rozzini L, Chilovi BV, Peli M, et al (2009) Anxiety symptoms in mild cognitive impairment. Int J Geriatr Psychiatry 24:300–305. https://doi.org/10.1002/gps.2106
91. Sacco R, Santangelo G, Stamenova S, et al (2016) Psychometric properties and validity of Beck Depression Inventory II in multiple sclerosis. Eur J Neurol 23:744–750. https://doi.org/10.1111/ene.12932
92. Sansone VA, Lizio A, Greco L, et al (2017) The Myotonic Dystrophy Health Index: Italian validation of a disease-specific outcome measure. Neuromuscul Disord 27:1047–1053. https://doi.org/10.1016/j.nmd.2017.07.004
93. Santangelo G, Morgante L, Savica R, et al (2009) Anhedonia and cognitive impairment in Parkinson’s disease: Italian validation of the Snaith-Hamilton Pleasure Scale and its application in the clinical routine practice during the PRIAMO study. Park Relat Disord 15:576–581. https://doi.org/10.1016/j.parkreldis.2009.02.004
94. Santangelo G, Sacco R, Siciliano M, et al (2016) Anxiety in Multiple Sclerosis: psychometric properties of the State-Trait Anxiety Inventory. Acta Neurol Scand 134:458–466. https://doi.org/10.1111/ane.12564
95. Santangelo G, Barone P, Cuoco S, et al (2014) Apathy in untreated, de novo patients with Parkinson’s disease: validation study of Apathy Evaluation Scale. J Neurol 261:2319–2328. https://doi.org/10.1007/s00415-014-7498-1
96. Santangelo G, Falco F, D’Iorio A, et al (2016) Anxiety in early Parkinson’s disease: Validation of the Italian observer-rated version of the Parkinson Anxiety Scale (OR-PAS). J Neurol Sci. https://doi.org/10.1016/j.jns.2016.06.008
97. Santangelo G, Raimo S, Siciliano M, et al (2017) Assessment of apathy independent of physical disability: validation of the Dimensional Apathy Scale in Italian healthy sample. Neurol Sci. https://doi.org/10.1007/s10072-016-2766-8
98. Santangelo G, Siciliano M, Trojano L, et al (2017) Apathy in amyotrophic lateral sclerosis: insights from Dimensional Apathy Scale. Amyotroph Lateral Scler Front Degener 18:434–442. https://doi.org/10.1080/21678421.2017.1313865
99. Siciliano M, Trojano L, Trojsi F, et al (2019) Assessing anxiety and its correlates in amyotrophic lateral sclerosis: The state-trait anxiety inventory. Muscle and Nerve 60:47–55. https://doi.org/10.1002/mus.26475
100. Solari A, Filippini G, Mendozzi L, et al (1999) Validation of Italian multiple sclerosis quality of life 54 questionnaire. J Neurol Neurosurg Psychiatry 67:158–162. https://doi.org/10.1136/jnnp.67.2.158
101. Spaccavento S, Craca A, Del Prete M, et al (2013) Quality of life measurement and outcome in aphasia. Neuropsychiatr Dis Treat 10:27–37. https://doi.org/10.2147/NDT.S52357
102. Spagnoli A, Foresti G, Macdonald A, et al (1987) Italian version of the organic brain syndrome and the depression scales from the CARE: Evaluation of their performance in geriatric institutions. Psychol Med 17:507–513. https://doi.org/10.1017/S003329170002506X
103. Tesio L, Cantagallo A (1998) The functional assessment measure (FAM) in closed traumatic brain injury outpatients: a Rasch-based psychometric study. J Outcome Meas 2:79–96
104. Tesio L, Alpini D, Cesarani A, Perucca L (1999) Short form of the dizziness handicap inventory: Construction and validation through Rasch analysis. Am J Phys Med Rehabil 78:233–241. https://doi.org/10.1097/00002060-199905000-00009
105. Tesio L, Granger C V., Perucca L, et al (2002) The FIMTM instrument in the United States and Italy: A comparative study. Am J Phys Med Rehabil 81:168–176. https://doi.org/10.1097/00002060-200203000-00003
106. Tofani M, Massai P, Fabbrini G, et al (2019) Psychometric properties of the italian version of the barthel index in patients with parkinson’s disease: A reliability and validity study. Funct Neurol 34:145–150
107. Tozzi V, Balestra P, Murri R, et al (2004) Neurocognitive impairment influences quality of life in HIV-infected patients receiving HAART. Int J STD AIDS 15:254–259. https://doi.org/10.1258/095646204773557794
108. Tremolizzo L, Lizio A, Santangelo G, et al (2020) ALS Cognitive Behavioral Screen (ALS-CBS): normative values for the Italian population and clinical usability. Neurol Sci 41:835–841. https://doi.org/10.1007/s10072-019-04154-1
109. Vellone E, Savini S, Fida R, et al (2015) Psychometric evaluation of the stroke impact scale 3.0. J Cardiovasc Nurs 30:229–241. https://doi.org/10.1097/JCN.0000000000000145
110. Verrusio W, Renzi A, Spallacci G, et al (2018) The development of a new tool for the evaluation of handicap in elderly: the Geriatric Handicap Scale (GHS). Aging Clin Exp Res 30:1187–1193. https://doi.org/10.1007/s40520-018-0907-z
111. Vignatelli L, Plazzi G, Barbato A, et al (2003) Italian version of the Epworth sleepiness scale: External validity. Neurol Sci 23:295–300. https://doi.org/10.1007/s100720300004
112. Voormolen DC, van Exel J, Brouwer W, et al (2021) A validation study of the CarerQol instrument in informal caregivers of people with dementia from eight European countries. Qual Life Res 30:577–588. https://doi.org/10.1007/s11136-020-02657-5
113. Zanetti O, Frisoni GB, Rozzini L, et al (1998) Validity of direct assessment of functional status as a tool for measuring Alzheimer’s disease severity. Age Ageing 27:615–622. https://doi.org/10.1093/ageing/27.5.
